# Activity of vitamin D receptor agonists against dengue virus

**DOI:** 10.1038/s41598-020-67783-z

**Published:** 2020-07-02

**Authors:** Janejira Jaratsittisin, Bin Xu, Wannapa Sornjai, Zhibing Weng, Atichat Kuadkitkan, Feng Li, Guo-Chun Zhou, Duncan R. Smith

**Affiliations:** 1 Institute of Molecular Biosciences, Mahidol University, Salaya, 73170 Thailand; 2 School of Pharmaceutical Sciences, Nanjing Tech University, Nanjing, 211816 Jiangsu China

**Keywords:** Microbiology, Virology, Antivirals

## Abstract

Infections with the mosquito-transmitted dengue virus (DENV) are a pressing public health problem in many parts of the world. The recently released commercial vaccine for DENV has encountered some problems, and there is still no effective drug to treat infections. Vitamin D has a well characterized role in calcium and phosphorus homeostasis, but additionally has a role in the immune response to bacterial and viral pathogens. In this study a number of fused bicyclic derivatives of 1*H*-pyrrolo[1,2]imidazol-1-one with vitamin D receptor (VDR) agonist activity were evaluated for possible anti-DENV activity. The results showed that five of the compounds were able to significantly inhibit DENV infection. The most effective compound, ZD-3, had an EC_50_ value of 7.47 μM and a selective index of 52.75. The compounds were only effective when used as a post-infection treatment and treatment significantly reduced levels of infection, virus output, DENV protein expression and genome copy number. These results suggest that these VDR agonists have the potential for future development as effective anti-DENV agents.

## Introduction

Infections with the mosquito transmitted dengue virus (DENV) have increased dramatically over the past few decades, and DENV is a significant public health problem in many tropical and sub-tropical countries^1^. Belonging to the genus *Flavivirus* within the family *Flaviviridae*, there are four distinct serotypes consisting of DENV serotype 1 to 4 (DENV 1–4). DENV has a positive sense, single stranded RNA genome that encodes for a single polyprotein that undergoes cleavage either by host and viral proteases into three structural proteins, capsid (C), pre-membrane (pr/M), envelope (E) and seven non-structural proteins (NS1, NS2A, NS2B, NS3, NS4A, NS4B, and NS5)^2^. Most cases of infection with DENV are asymptomatic^3^, although infection can result in a mild self-limited febrile illness called dengue fever (DF). In some cases more severe symptoms can resulting in dengue hemorrhagic fever (DHF) or dengue shock syndrome (DSS) which is characterized by significant complications such as plasma leakage, internal bleeding, organ failure, and shock^3^. Currently, there is no specific treatment for DENV. While a commercial vaccine is available in some DENV endemic countries, there are controversies about the protection efficiency among different DENV serotypes^4^, as well as the occurrence of more severe disease when *Flavivirus* naïve individuals receive vaccination^5^. While several drugs have entered small scale trials^6,7^ most of the candidate drugs have failed to reduce the viremia of the participants.

Over the past decade host micronutrients status and micronutrient supplementation have been widely studied with regards to their potential antiviral activity, including the fat-soluble vitamin D, which has a major role in calcium and phosphorous homeostasis^8^. Vitamin D is obtained from the two major sources namely the diet or through UV-mediated synthesis initiated in the skin^8^. The biologically active form of vitamin D or calcitriol (1,25(OH)_2_D_3_) requires the two sequential hydroxylation reactions at the liver and kidney, respectively^9^. 1,25(OH)_2_D_3_ binds to vitamin D receptor (VDR) which is a nuclear receptor superfamily and a ligand activated transcription factor^10^. Subsequently vitamin D/VDR form a complex with retinoid X receptors (RXR) and is translocated into the nucleus to bind with specific vitamin D response elements (VDREs). Depending on the target genes, either co-activators or co-repressors are attracted to the complex to induce or repress RNA polymerase II-mediated gene transcription^11^.

In addition to the regulation of calcium metabolism, vitamin D/VDR is also involved in several biological actions including cell differentiation, proliferation and immunomodulation^10^. Vitamin D activates both innate and adaptive immune response through several mechanisms including T-cells activation, macrophage differentiation and the production of anti-microbial peptides such as cathelicidin (LL-37) and β-Defensin^12–14^. Vitamin D deficiency has been shown to induce increased susceptibility to viral infections including hepatitis C virus, influenza virus and HIV^15–17^. However, the association between vitamin D/VDR and dengue infection is not completely understood. However, it has been shown that there is a relationship between vitamin D levels and VDR polymorphism and the severity of DENV clinical manifestation^18,19^. Treatment of DENV infected monocytic U937 cells or hepatic Huh-7 cells with 1,25(OH)_2_D_3_ resulted in decreased numbers of infected cells, reduced Toll-like receptors and lowered inflammatory cytokines^20^. Another study demonstrated that the presence of 1,25-dihydroxyvitamin D3 during macrophage proliferation restricted DENV infection and altered the proinflammatory cytokine response through reducing the expression of the C-type lectin mannose receptor, a DENV receptor protein^21^. In a recent study a novel class of VDR agonists were described^22^ and this study sought to determine if these compounds had antiviral effects.

## Results

### Evaluation of cytotoxicity of VDR agonists

Prior to determining possible anti-DENV activity of the newly reported VDR agonists^22^, the cytotoxicity of the compounds to HEK293T/17 cells was determined by trypan blue staining and by the MTT assay. Additionally cell morphology was evaluated by observation under an inverted microscope. The trypan blue exclusion assay showed little cytotoxicity at concentrations up to 200 μM (Supplemental Figure S1A), while the MTT assay showed a dose dependent cytotoxicity (Supplemental Figure S1B). The calculated CC_50_ values are shown in Table 1. Additionally observation of cell morphology showed signs of morphological changes at higher concentrations (Supplemental Figure S2). Based on all the data, further VDR agonist treatments were carried out with a concentration of 10 μM.

**Table 1:** General information of vitamin D receptor agonists.

Compound ID	Chemical formula	Formula weight	CC_50_ (µM)	EC_50_ (µM)	Selective index (SI)
ZD-1	C_16_H_15_N_3_O_4_	313.30	259.68	8.74	29.71
ZD-2	C_19_H_19_N_3_O_5_	369.37	313.71	11.77	26.65
ZD-3	C_23_H_27_CIN_4_O_5_S	506.13	394.06	7.47	52.75
ZD-4	C_28_H_35_N_5_O_6_S	569.23	519.58	33.45	15.53
ZD-5	C_21_H_23_N_3_O_5_	397.42	438.62	8.46	51.84
ZD-6	C_18_H_17_N_3_O_4_	339.12	276.92	11.32	24.46
ZD-20	C_28_H_34_N_4_O_7_S	570.21	908.29	151.72	5.98

**Figure 1:**
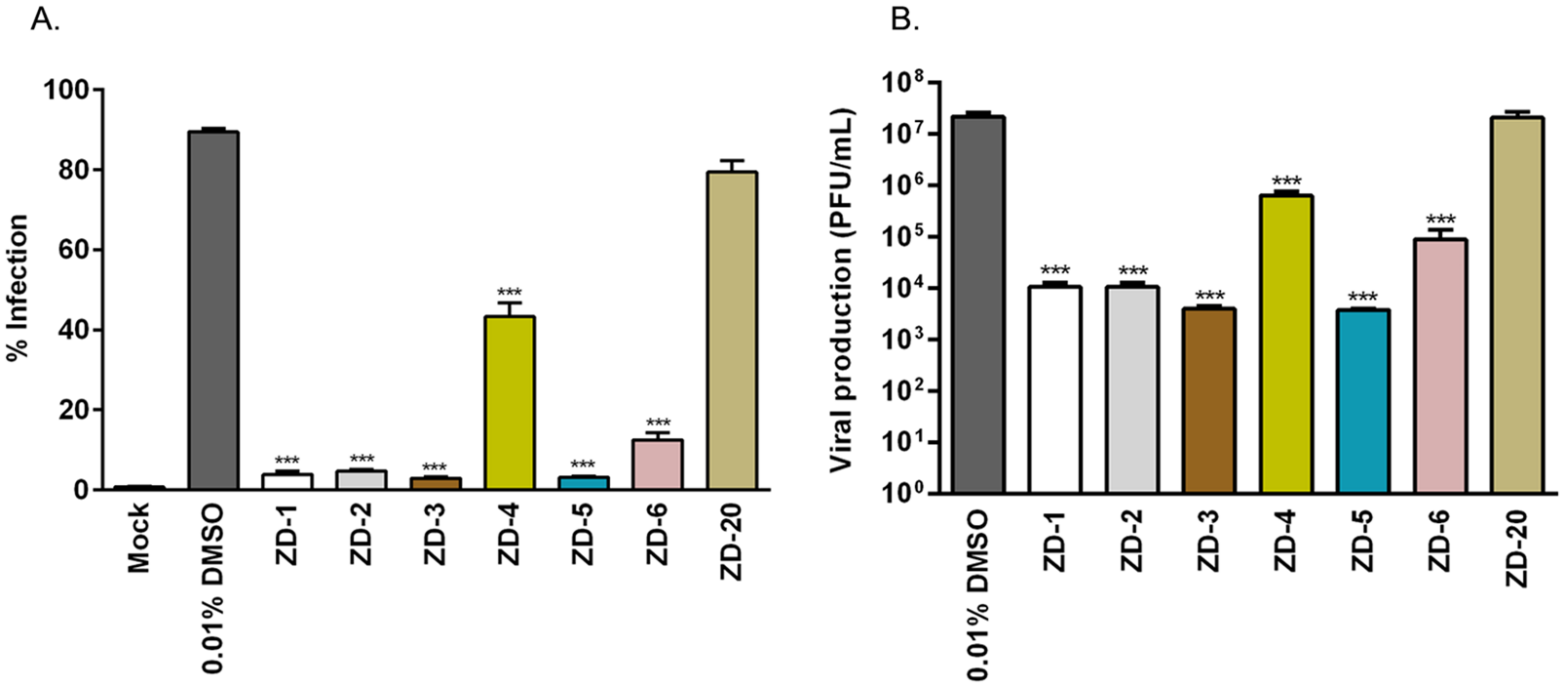
Antiviral activity of VDR agonists. Mock and DENV 2 infected HEK293T/17 cells were treated with 0.01% DMSO or 10 µM of ZD-1, ZD-2, ZD-3, ZD-5, ZD-6 and ZD-20. (**A**) At 24 h.p.i. the treated cells were collected and the levels of infection determined using flow cytometry. (**B**) In parallel, the supernatants of the treated cells were evaluated for virus titer by standard plaque assay. p value; *< 0.05, **< 0.01, ***< 0.001 for significance. The experiment was performed independently in triplicate with duplicate in plaque assay.

Next, the effect of the seven VDR agonists on DENV infection was evaluated. HEK293T/17 cells were mock-infected or infected with DENV 2, followed by the incubation with 10 µM VDR agonists. After 24 h of treatment, the level of infection was determined by flow cytometry. Results (Fig. 1) showed that ZD-1, ZD-2, ZD-3, ZD-5, and ZD-6 treated cells showed highly significant reductions in the level of infection. For the remaining two compounds, treatment with ZD-4 resulted in infection levels being reduced by approximately 50%, while treatment with ZD-20 showed no significant reduction in the level of infection as compared to the control of DENV 2 infected cells treated with 0.01% DMSO control (Fig. 1A). Evaluation of the DENV 2 titer in the supernatant by standard plaque assay showed that treatment with ZD-1, ZD-2, ZD-3, ZD-5, and ZD-6 reduced virus production by 2-3Log_10_, while treatment with ZD-4 reduced virus output by slightly over 1Log_10_. Again, no significant effect was observed with treatment with ZD-20 (Fig. 1B). Overall there was consistency in the reductions seen in level of infection and final virus titer for all compounds.

Given the significant reductions seen in virus production for the majority of compounds, we next determined the half-maximum effective concentration (EC_50_) and selective index (SI) for all compounds. HEK293T/17 cells were mock-infected or infected with DENV 2, followed by incubation with various concentrations (0.1 nM to 10 µM for ZD-1,-2,-3,-5, and -6, and or 1–100 µM for ZD-4 and -20) of all VDR agonists. At 24 h post-treatment, virus titer in the supernatant was determined by plaque assay. Results (Supplemental Figure S3 and Table 1) showed that the EC_50_ values ranged from 7.47 μM (ZD-3) to 151.72 μM (ZD-20) and the selective index values ranged from 52.75 (ZD-3) to 5.98 (ZD-20). ZD-1, ZD-2, ZD-3, ZD-5, and ZD-6 which showed the greatest antiviral effects also showed the highest SI values (Table 1). These five VDR agonists were therefore selected for further investigation.

### Evaluation of virucidal activity of VDR agonists

To determine whether the compounds possessed direct virucidal activity, stock DENV 2 was directly incubated with 10 µM of VDR agonists at 37 °C for 1 h, after which the titer was determined by standard plaque assay. Results (Supplemental Figure S4) showed no reduction of virus titer after the incubation compared to the DMSO control, showing that the VDR agonists have no direct virucidal activity**.**

### Effect of time-of-additional of VDR agonists

**Figure 2:**
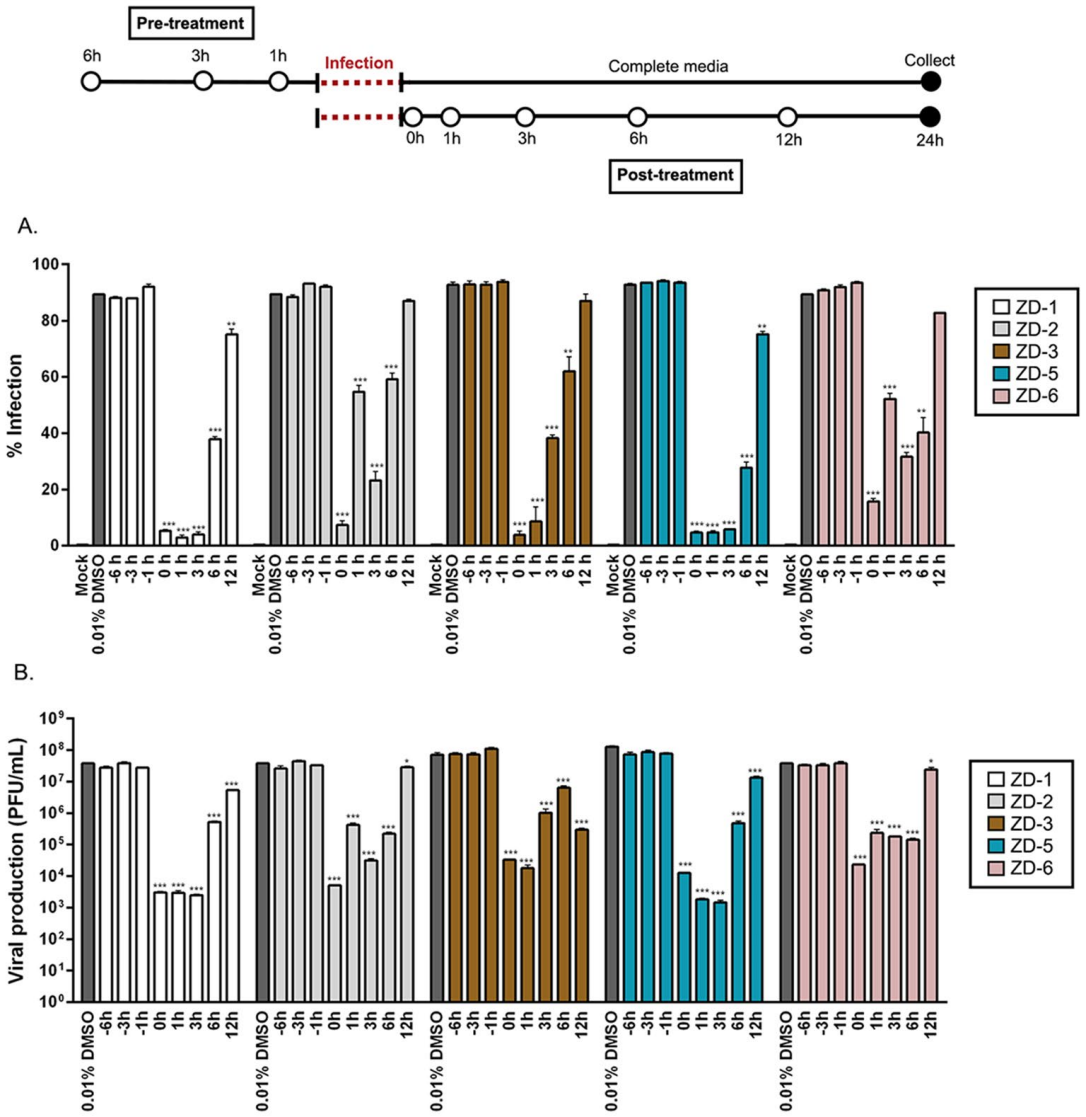
Time-of-additional of VDR agonists. Mock or DENV infected HEK293T/17 cells were pre- and post-treated with 0.01% DMSO and 10 µM of ZD-1, ZD-2, ZD-3, ZD-5 and ZD-6 at various time points as shown in the diagram. (**A**) At 24 h.p.i. the treated cells were collected and the levels of infection determined using flow cytometry. (**B**) In parallel, the supernatant of the treated cells were evaluated for virus titer by standard plaque assay. p value; *< 0.05, **< 0.01, ***< 0.001 for significance. The experiment was performed independently in triplicate with duplicate plaque assay.

To assess the effect of VDR agonist treatment at a different time points during infection, both pre- and post-infection treatment was evaluated. In pre-treatment cells were treated with 10 µM of the five most effective VDR agonists (ZD-1, ZD-2, ZD-3, ZD-5, and ZD-6) for 6, 3 or 1 h before infection, and after 24 h both the level of infection and the virus titer in the supernatant were determined by flow cytometry and standard plaque assay respectively. In addition, cells were collected at the end of the experiment for subsequent protein isolation, while the remaining supernatant was used to determine genome copy number. For post-infection treatment, cells were infected with DENV 2 before being treated with the 5 VDR agonists at 0, 1, 3, 6 and 12 h post-infection (with 0 h representing treatment with the compounds immediately after the end of the infection step), and at 24 h post-infection the level of infection and the virus titer in the supernatant were again determined by flow cytometry and standard plaque assay respectively. Again, cells were collected at the end of the experiment for subsequent protein isolation, while the remaining supernatant was used to determine genome copy number. The results (Fig. 2) showed that the compounds had no effect when administered pre-infection on either level of infection or virus titer in the supernatant. Post-infection treatment of the compounds exerted considerable effects on both the level of infection and on the virus titer (Fig. 2), with post-infection treatment resulting in highly significant reductions in both level of infection and virus titer. The largest effect was observed with treatment immediately after the infection step, and for up to 3 h post-infection, however, significant reductions in both the level of infection and virus titer were observed at 6 and 12 h post-infection treatment with compounds ZD-1 and ZD-5 (Fig. 2).

**Figure 3:**
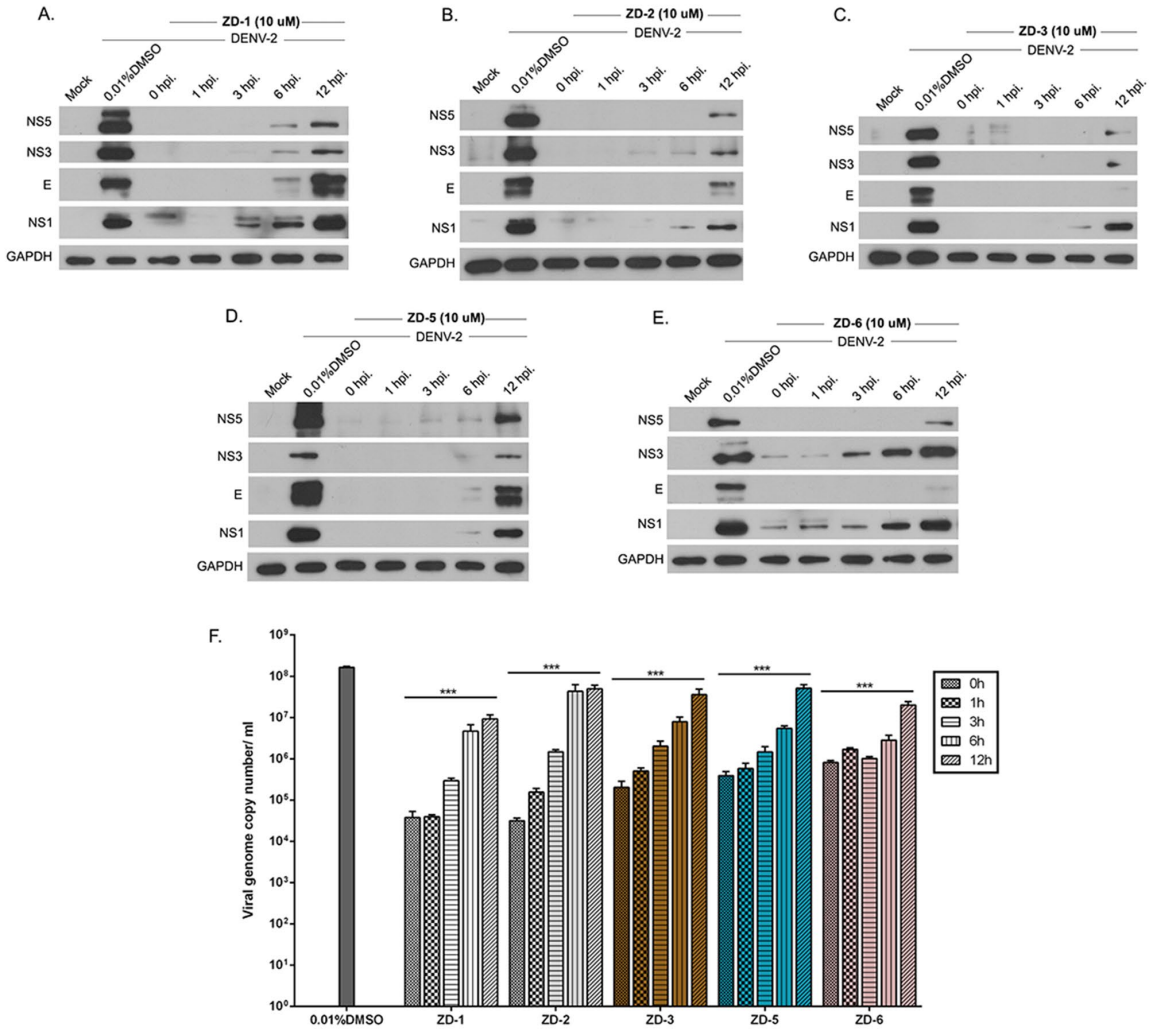
VDR agonist activity on viral protein and viral genome copy number. (**A**–**E**) HEK293T/17 cells were mock or DENV 2 infected followed by treatment with 0.01% DMSO or 10 µM of VDR agonists (ZD-1, ZD-2, ZD-3, ZD-5 and ZD-6) at the indicated time points. Expression of DENV structural protein (**E**) and non-structural proteins (NS1, NS3 and NS5) was determined by western blot assay, using GAPDH as an internal control. White spaces separate different antibody probing. (**F**) In parallel, the supernatant were collected the level of the viral genome copy number determined by qRT-PCR. Viral genome copy number was calculated by comparing with tenfold serial dilution of a DENV NS1 standard control. p value; *< 0.05, **< 0.01, ***< 0.001 for significance. All experiments were performed independently in triplicate.

Proteins from the post-treatment experiments were used to determine the expression level of DENV 2 structural (E protein) and non-structural (NS1, NS3, and NS5) proteins by western blotting while RNA from the supernatant was used to establish DENV genome copy number by real-time RT-PCR. Results (Fig. 3) showed that expression of both structural and non-structural proteins was largely completely absent when treatment occurred at an early time point in infection, although reduced protein expression could be observed in cells treated with the VDR agonists as long as 12 h post-infection. Similarly, while large reductions were seen in the level of DENV genome in the supernatant (consistent with the plaque assay results), smaller, but still statistically significant results were seen in cells treated with all VDR agonists as late as 12 h post-infection (Fig. 3F).

### VDR agonist activity as assessed by an immunofluorescence assay

**Figure 4:**
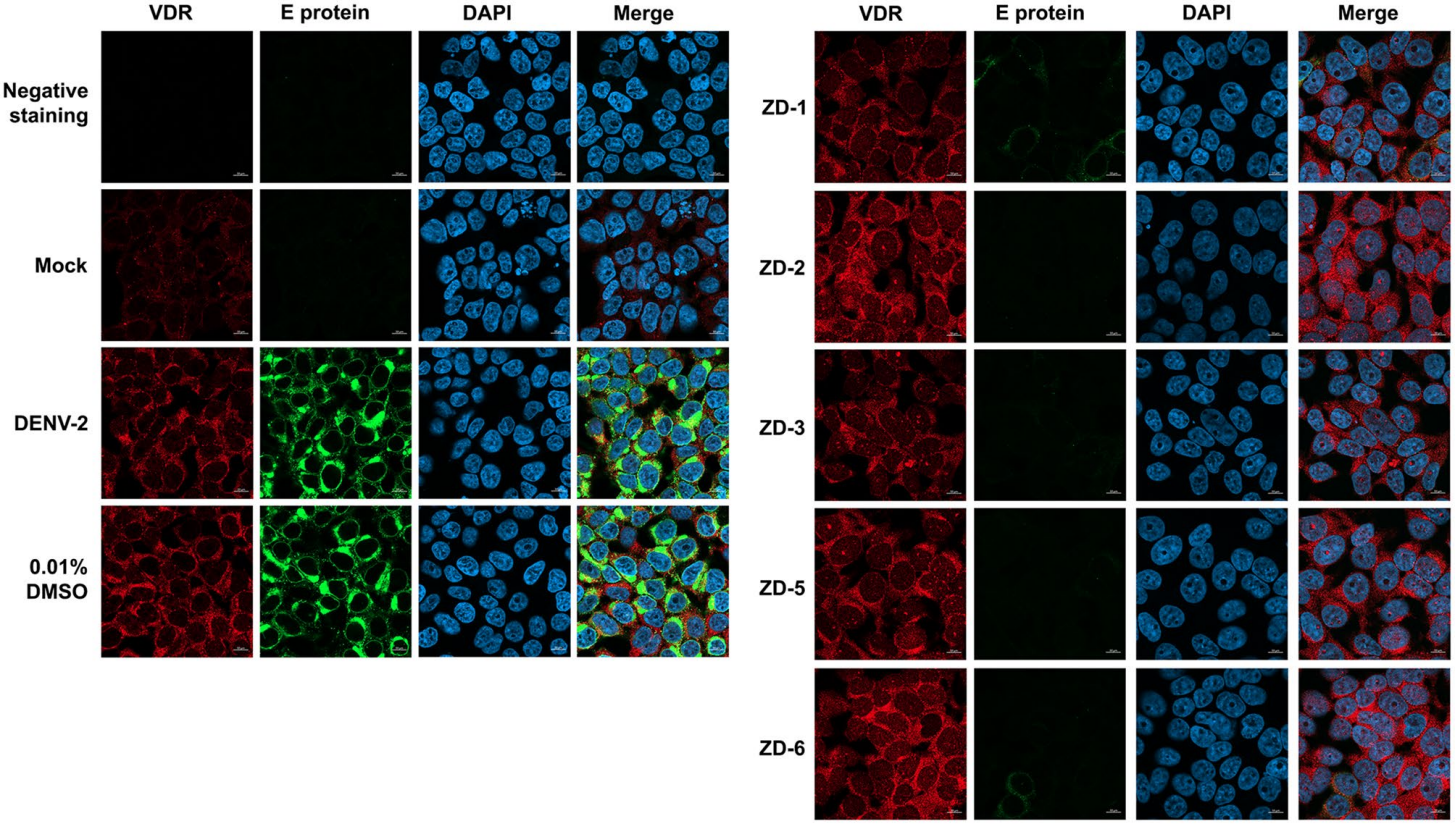
VDR agonist activity on DENV E protein and VDR expression assessed by immunofluorescence assay. Mock or DENV 2 infected HEK293T/17 cells were treated with 0.01% DMSO or 10 μM of VDR agonists (ZD-1, ZD-2, ZD-3, ZD-5 and ZD-6). After 24 h of treatment, cells were processed under the standard procedure of immunofluorescence assay. DENV E protein (green) and VDR (red) were detected using specific antibodies. Nuclei were stained with DAPI (blue). All the signal was observed under a LSM 800 w Airyscan (ZEISS, Oberkochen, Germany) confocal microscope with 60X magnification.

To confirm the results of the earlier experiments, an immunofluorescence assay was performed. Mock infected and DENV 2 infected HEK293T/17 cells grown on coverslips were treated with 10 µM of the five VDR agonists for 24 hpi, after which the expression of DENV E protein and VDR detected after incubation with specific antibodies and examination under a confocal microscope. The results (Fig. 4) were consistent with the earlier observations, with DENV E protein being largely undetectable after treatment with the VDR agonists. Interestingly, cells infected with DENV 2 showed a markedly higher signal for VDR than mock infected cell, and apparently higher expression was observed in VDR agonist treated cells (Fig. 4). It is known that the binding of a ligand to the VDR can induce translocation of VDR into the nucleus, and the results appeared to be consistent with the increased localization of VDR to the nucleus in agonist treated cells. The co-localization between VDR and nuclear staining DAPI was captured using Airyscan with comparison between mock-infected, DENV 2 infected and DENV 2 infected and ZD-6 treated cells (Supplemental movie 1A–C). In combination the results support that the VDR agonists can induce the expression of VDR and also activate its nuclear translocation.

### Evaluation of cell type specificity

**Figure 5:**
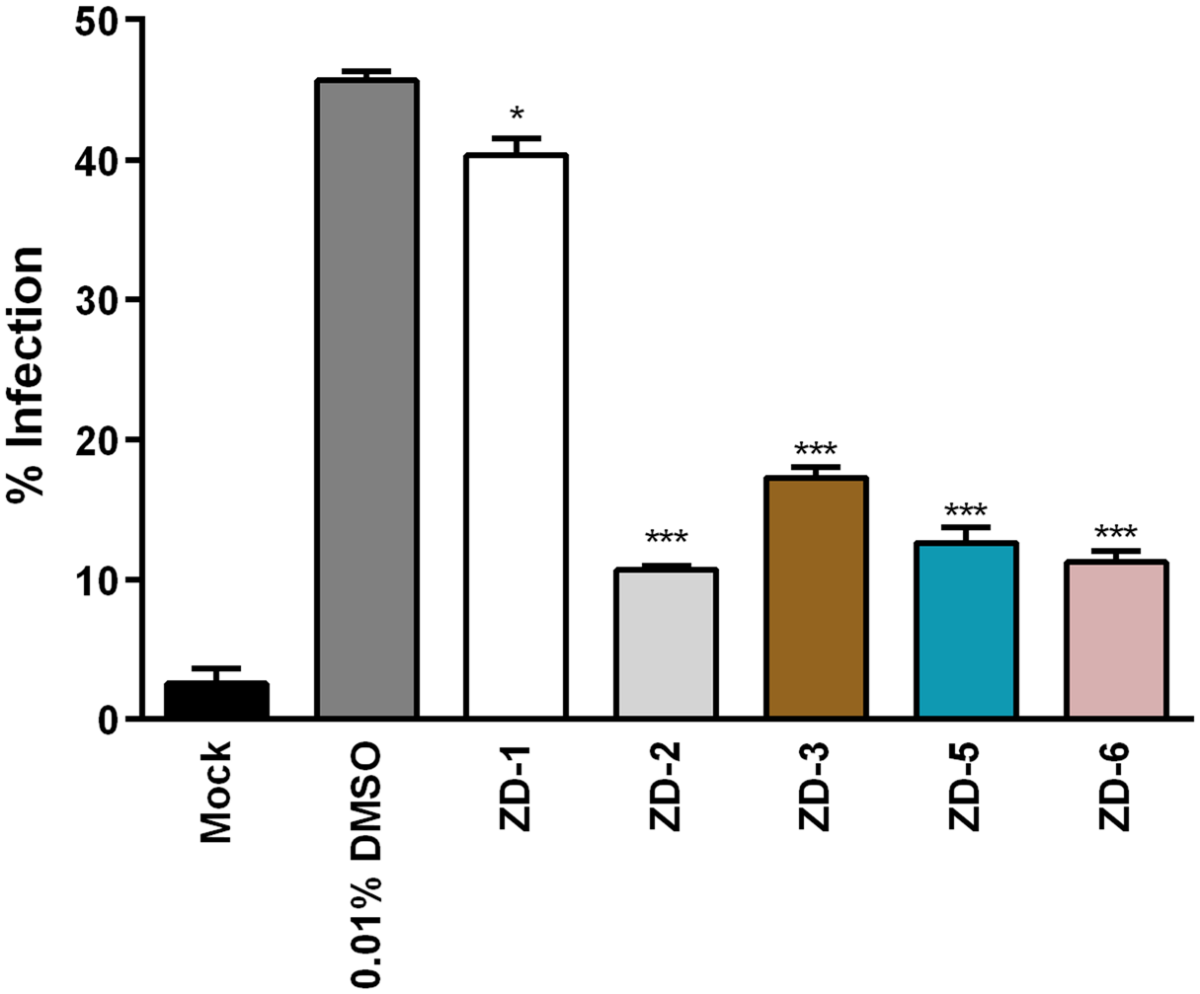
Antiviral activity of VDR agonists against DENV infection on HepG2 cells. HepG2 cells were mock-infected or infected with DENV 2 at MOI 5 for 2 h, followed by the treatment with 10 μM of ZD-1, ZD-2, ZD-3, ZD-5 and ZD-6 for 24 h.p.i. The treated cells were collected and level of infection determined by flow cytometry. p value; *< 0.05, **< 0.01, ***< 0.001 for significance. The experiment was performed independently in triplicate.

Given that HEK293T/17 cells are not representative of a DENV target tissue, the antiviral activity of the VDR agonists was evaluated in cells representative of a primary target cell. For this reason, HepG2 cells were infected with DENV 2, followed by treatment with 10 μM of the five effective VDR agonists (ZD-1, ZD-2, ZD-3, ZD-5, and ZD-6) for 24 h, after which the level of infection was determined by flow cytometry. Results (Fig. 5) showed a significant reduction in infection for all compounds tested, albeit that the reduction seen with ZD-1 was modest as compared to the other four compounds.

### Activity of VDR agonists against other flaviviruses

**Figure 6:**
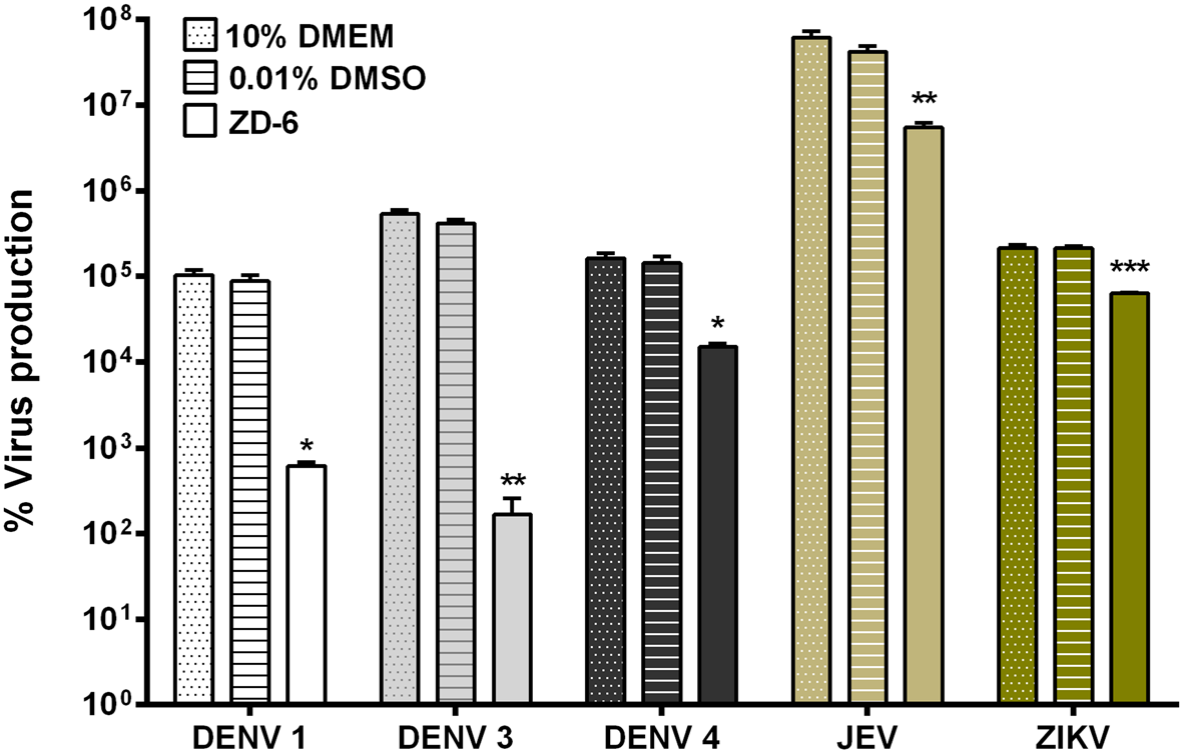
Antiviral activity of a VDR agonist against other flaviviruses. HEK293T/17 cells were infected with other flaviviruses, including DENV 1 (strain 16007), DENV 3 (strain 16562), DENV 4 (strain 1036), JEV (strain BJ1) and ZIKV (strain SV0010/15). Infected cells were incubated with 10 μM ZD-6 in complete medium and at 24 h.p.i., virus titer was determined by standard plaque assay. p value; *< 0.05, **< 0.01, ***< 0.001 for significance. The experiment was performed independently in triplicate with duplicate in plaque assay.

The broad spectrum anti-flavivirus activity of the VDR agonists was initially investigated with only ZD-6. HEK293T/17 cells were infected with DENV 1, 3, 4, JEV and ZIKV. The infected cells were incubated with 10 μM of ZD-6 for 24 h, after which the virus titer in the supernatant was determined by standard plaque assay. The results (Fig. 6) showed that treatment with ZD-6 significantly inhibited production of all the viruses investigated. The magnitude of inhibition varied between 1–3 Log_10_, with the lowest effects being seen for JEV and ZIKV.

### Agonist activity of VDR agonists

**Figure 7:**
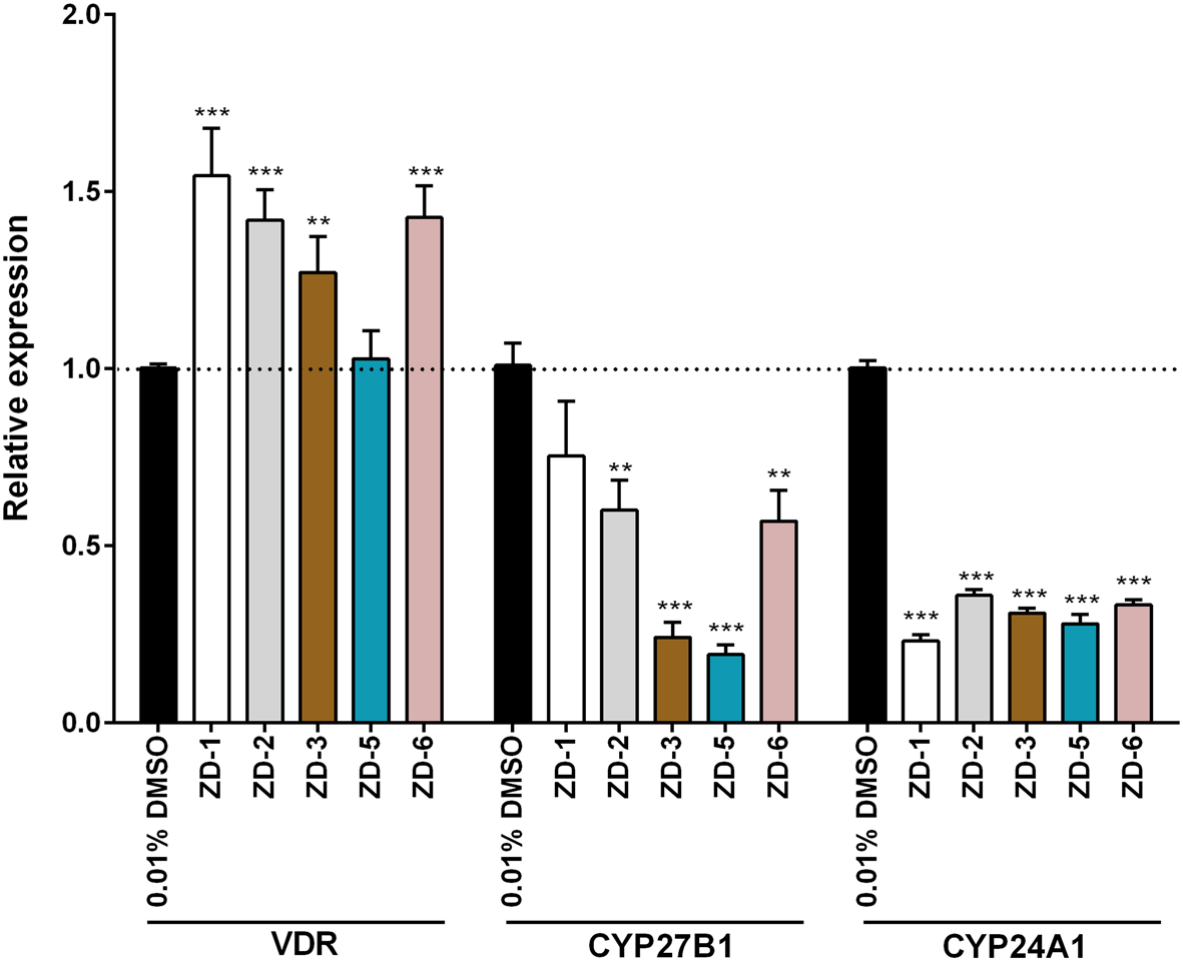
VDR regulated gene expression by qRT-PCR. HEK293T/17 cells were treated with 10 µM VDR agonists for 24 h. RNA was isolated and gene expression levels determined using qRT-PCR. The relative gene expression of VDR, CYP27B1 and CYP24A1 was normalized against a housekeeping gene (actin) and untreated cells (0.01%DMSO), respectively. p value; *< 0.05, **< 0.01, ***< 0.001 for significance. All experiments were performed in triplicate.

Upon translocation to the nucleus the VDR mediates gene transcription to induce the expression of CYP24A1 that functions to degrade active vitamin D, while suppressing the expression of CYP27B1 which functions to synthesize active vitamin D^23^. We therefore investigated the transcriptional response of VDR, CYP24A1 and CYP27B1 to the five VDR agonists investigated in this study. Results show that four of the five VDR agonists significantly increased expression of VDR, and all reduced expression of CYP27B1 (Fig. 7). Interestingly, all agonists also significantly suppressed expression of CYP24A1 (Fig. 7), in contrast to the natural action of vitamin D.

### Evaluation of a commercial VDR agonist

**Figure 8:**
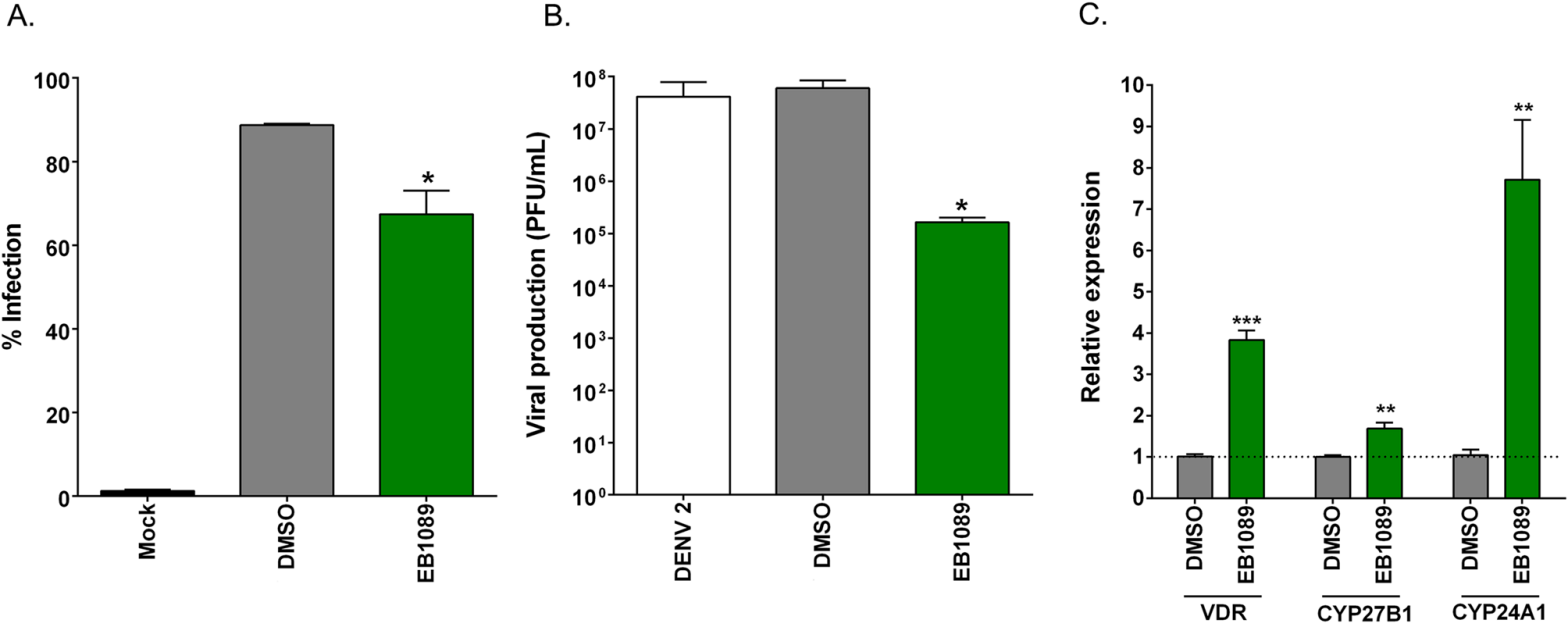
The antiviral and agonist activity of commercial VDR agonist EB1089. (**A**, **B**) Mock and DENV 2 infected HEK293T/17 cells were treated with 20 µM EB1089 for 24 after which (**A**) the level of infection was determined by flow cytometry and (**B**) the virus titer in the supernatant was determined by standard plaque assay. (**C**) HEK293T/17 cells were treated with 20 µM EB1089 or DMSO vehicle for 24 h, after which cells were collected and RNA extracted and gene expression of VDR, CYP27B1, CYP24A1 and actin determined by qRT-PCR. The relative gene expression of VDR, CYP27B1 and CYP24A1 was normalized against actin and untreated cells (0.02% DMSO), respectively. p value; *< 0.05, **< 0.01, ***< 0.001 for significance. All experiments were performed in triplicate, with duplicate plaque assay where appropriate.

We additionally evaluated the activity of a commercial vitamin D analog, EB1089. HEK293T/17 cells were infected with DENV 2, followed by treatment with 20 μM EB1089. At 24 h.p.i., the treated cells and supernatant were collected to examine the levels of infected cell and the viral production, respectively. Treatment of EB1089 significantly reduced the levels of both infected cells and viral yield (Fig. 8A,B). Viral production was reduced by approximately 2Log_10_, consistent with the activity seen for the VDR agonists examined in this study.

In addition, the regulation of VDR-mediated gene expression was evaluated by the incubation of HEK293T/17 cells with 20 µM EB1089 for 24 h. The relative gene expression was examined determined by qRT-PCR and normalized against a house keeping gene (actin) and a DMSO control. The transcriptional levels of VDR, CYP27B1 and CYP24A1 were significantly up regulated after treatment with EB1089 (Fig. 8C). As expected, EB1089 which an analog of 1,25-dihydroxyvitamin D3, successfully mimicked the action of active vitamin D3 by inducing the expression of CYP24A1, in contrast to the lack of induction of this gene seen with the VDR agonists used in this study.

## Discussion

Finding specific and efficient anti-viral drugs is proving to be a challenge for DENV researchers. Host nutritional status and nutritional supplementation such as vitamin D, is one possible strategy for anti-DENV drug development^14^. Vitamin D exerts its activity through binding to VDR. In addition to having a role in calcium homeostasis^8^, vitamin D also plays a role in several biological systems, including the immune response and pathogen-defense mechanisms^10^. Prior studies have shown the antiviral activity of vitamin D against Flaviviruses including hepatitis C virus^15,17,24,25^ and DENV^18,20,21,26,27^.

A recent study identified fused bicyclic derivatives of 1*H*-pyrrolo[1,2]imidazol-1-one as potent regulators of VDR signaling^22^, and in this study a number of these compounds were shown to act as potent inhibitors of DENV infection. In a previous study it was proposed that vitamin D exerted its antiviral activity at the step of viral entry, as human macrophages differentiated in the presence of vitamin D showed reduced expression of the DENV receptor protein the C-type lectin mannose receptor^21^. However, this is not consistent with the results seen in this study as the most significant effect was seen with the compounds added immediately after the infection step, and for up to three hours post infection, while pre-treatment of cells with the VDR agonists had no effect on infection.

In monocytes, vitamin D has been proposed to regulate slightly less than 200 different genes to different extents^28^, and it is likely that similar numbers of genes are regulated in different cell types. Thus, knowing how the VDR agonists are specifically exerting their antiviral effect is going to be difficult. In their study Xu et al.^22^ showed that the VDR agonists when administered together with calcitrol activated p62 expression as well as changed the LC3-II/LC3-I ratio, suggestive of the induction of autophagy. However, autophagy in DENV infection is generally proviral^29–31^, and as such the agonists are unlikely to be exerting their effect through induction of autophagy.

Previously Hass et al.^32^ have shown that Vitamin D can inhibit endoplasmic and oxidative stress^32^. Several studies have shown that ER stress is activated during DENV infection^33–35^, and studies have suggested that this activation is essential for DENV replication^33,35^. Hass et al.^32^ additionally demonstrated that vitamin D could down regulate the ER stress induced up-regulation of GRP78. Several studies have shown the up-regulation of GRP78 in DENV infection^36–38^, and that this protein is essential for DENV replication^36,38^. Thus the VDR agonists exerting their effect through modulation of ER stress could have a major effect on DENV replication as observed here.

Typically, active vitamin D (1,25-dihydroxy vitamin D3) is generated by the action of 1,25-dihydroxylase (CYP27B1) in the kidney. Binding of active vitamin D to the VDR results in translocation to the nucleus where it can bind to specific vitamin D responsive elements (VDRE). These complexes can recruit transcription factors to mediate gene transcription^23^. As part of the transcriptional response, 24-hydroxylase, a catabolic enzyme for vitamin D that is encoded CYP24A1 is induced, and CYP27B1 is suppressed and VDR can also regulate transcription of the VDR gene^23^. The agonists investigated in this study all increased the transcription of VDR, and suppressed transcription of CYP27B1 (the enzyme responsible for active vitamin D synthesis) but markedly all also suppressed transcription of CYP24A1, the enzyme involved in catabolism of vitamin D. However, a commercial vitamin D agonist showed elevated expression of CYP241A, and thus, it is likely that the agonists used here would have a longer effective time than vitamin D itself, and this would be supported by the more than 3Log_10_ inhibition seen with the agonists investigated in this study as compared with the approximately 2Log_10_ inhibition seen with the commercial agonist.

The cell line used in this study, HEK293T/17 is derived from human embryonic kidney^39^, and although the kidney is not believed to be a target organ for DENV infection, the cell line is widely used in DENV studies. In this case the use of HEK293T/17 with a functioning VDR response pathway has been useful in highlighting some of the mechanism of action of the agonists used here. However, examination of the effect of the agonists in HepG2 cells also showed an effect, suggesting that the drug has broad cell specificity. Markedly, the agonists used here additionally had effects (albeit to a lesser extent) on other flaviviruses including JEV and ZIKV, although the strongest effects were seen with DENV, both for DENV 2, and other DENV serotypes.

Vitamin D insufficiency and deficiency are a global problem, with high levels in America (36% of the general population), Northern Europe (92% of the general population), Asia (45–99% of the general population), North Africa (60% of the general population), as well as in Canada, the Middle East and Australia^40^. Thus, it is likely that most people living in dengue endemic areas are vitamin D deficient, thus vitamin D deficiency may increase the presentation of the disease. However, clinical evidence on vitamin D status during DENV infection remains scant. An investigation into serum vitamin D levels found that levels were higher in DF and DHF patients as compared to normal controls^18^, while a much larger recent study found no difference in 25-hydroxy vitamin D levels between controls and cases^41^. However, one study suggested that a combination of oral calcium carbonate and vitamin D3 improved the clinical presentation and reduced signs and symptoms of dengue fever^42^, although the number of patients was extremely low.

## Conclusion

In this study seven fused bicyclic derivatives of 1*H*-pyrrolo[1,2]imidazol-1-one with vitamin D receptor (VDR) agonist activity were evaluated for possible anti-DENV activity. The results showed that five of the compounds were able to significantly inhibit DENV infection. The most effective compound, ZD-3, had an EC_50_ value of 7.47 μM, a selective index of 52.75 and reduced virus production by more than 3Log_10_. These results suggest that these VDR agonists have the potential for future development as effective anti-DENV agents, and possibly as more broad-spectrum anti-flaviviral agents**.**

## Materials and methods

### Cell line and viruses

Human embryonic kidney HEK293T/17 (ATCC No. CRL-11268) and human hepatocellular HepG2 (ATCC No. HB-8065) cells were cultured in complete medium, composed of Dulbecco’s modified Eagle medium (DMEM; Gibco BRL) supplemented with 10% heat-inactivated fetal bovine serum (FBS) at 37 °C with 5% CO_2_. Dengue virus serotype 2 (DENV 2) strain 16681, DENV 1 (strain 16007), DENV 3 (strain 16562), DENV 4 (strain 1036), ZIKV (strain SV0010/15) and JEV (strain BJ1) were all propagated in the *Aedes albopictus* cell line C6/36 (ATCC No. CRL-1660). Viral progenies in the supernatant were collected and centrifuged at 1,000*g* to remove cell debris. The virus stocks were kept at − 80 °C until used. Virus titers were determined by standard plaque assay on LLC-MK2 cells (ATCC No. CCL-7).

### Vitamin D receptor (VDR) agonists

The seven VDR agonists (ZD-1, ZD-2, ZD-3, ZD-4, ZD-5, ZD-6, ZD-20) used in this study were as previously described^22^. General information, including compound ID, chemical formula and formula weight, is provided in Table 1. Chemical structures are given in Supplemental Table S1. In addition, the commercial VDR agonist, EB1089 (Tocris Cookson Ltd., Bristol, UK) was also used in this study. All compounds were dissolved with 100% DMSO to a final concentration of 100 mM and kept at − 30 °C until used.

### Cell viability assessment by cell morphology alteration, trypan blue staining, and MTT assay

To assess the toxicity of the VDR agonists, HEK293T/17 cells were cultured in 6-well plates until 70% confluence was obtained under standard conditions. The medium was replaced with the VDR agonists diluted with complete medium to various concentrations (1–200 µM). After 24 h of incubation, the cell morphology of the treated cells was observed under an inverted microscope, following which the treated cells were trypsinized and stained with 0.4% trypan blue solution. Cells were counted using a hemocytometer and the percentage cell viability determined.

To determined cell viability by the MTT assay (Thermo Fisher Scientific Inc., Waltham, MA), HEK293T/17 cells were cultured for 24 h on 96-well plates until 70% confluence was reached under the standard conditions. The cell culture medium was incubated with 100 µl of various concentrations of VDR agonists (1–800 µM) diluted with complete DMEM in parallel with DMSO control. After 24 h of incubation, MTT dye was added into each well and the formazan precipitant was dissolved with DMSO. Optical density at 570 nm was determined using a standard microplate reader. The percentage of cell viability was calculated from the average measurement of four replicates as compared with the negative control (cells treated with complete medium).

### Virucidal assay

Stock DENV 2 was incubated directly with medium only or with DMSO control or with 10 µM of VDR agonists in a final volume of 100 µl for 1 h at 37 °C, after which infectious virus titer was determined by standard plaque assay, essentially as described elsewhere^43^. All experiments were undertaken independently in triplicate with duplicate plaque assay.

### VDR agonist treatment

HEK293T/17 cells were cultured on 6-well plates until 70% confluence was reached under standard conditions. The cells were mock-infected or infected with DENV 2 at a multiplicity (MOI) of 5 for 2 h. After infection, the medium was removed and the infected cells were incubated with appropriate concentrations of the VDR agonists until 24 h.p.i., in parallel with DMSO treated control cells. Experiments were performed independently triplicate.

For time course experiments, under conditions of pre-infection treatment, HEK293T/17 cells were pre-treated with an appropriate concentration of VDR agonists for 1, 3 or 6 h before infection, followed by incubation in complete medium. For treatment post-infection, infected cells were treated with VDR agonists at 0, 1, 3, 6 and 12 h.p.i. The infected cells and supernatant for both pre- and post-treated samples were collected at 24 h.p.i. Experiments were performed independently triplicate.

For viruses other than DENV 2, HEK293T/17 cells were infected with DENV 1 (strain 16007), DENV 3 (strain 16562), DENV 4 (strain 1036) and ZIKV (strain SV0010/15) at MOI 10 and with JEV (strain BJ1) at MOI 1. At 2 h.p.i., the virus was removed and the cells were incubated with 10 μM of VDR agonist (ZD-6) in complete medium for 24 h, after which the supernatant was collected, and the virus titer was determined by standard plaque assay. Experiments were performed independently triplicate.

### Determination of viral infectivity by flow cytometry

HEK293T/17 cells were mock-infected or infected with DENV 2 at MOI 5 for 2 h, followed by treatment with the appropriate concentration of VDR agonists. At 24 hpi, the treated cells were harvested and Fc receptors were blocked with 10% goat serum (Gibco BRL, Gaithersburg, MD) for 30 min at 4 °C. The cells were washed with 1% BSA in 1X PBS/IFA (150 mM NaCl, 50 mM NaH_2_PO_4_, 50 m M Na_2_HPO_4_, pH to 7.4) and centrifuged at 6,000 g for 5 min. Then, the cells were fixed with 4% paraformaldehyde in 1X PBS/IFA at room temperature in the dark for 20 min, followed by the washing. Cell membrane permeabilization was performed using 0.2%Triton-X in 1X PBS/IFA for 10 min in the dark followed by overnight incubation with a 1:150 dilution of a pan-specific mouse monoclonal anti-dengue E protein antibody (HB114^44^). Cells were subsequently incubated for 1 h in the dark with a 1:40 dilution of a fluorescein isothiocyanate (FITC)-conjugated goat anti-mouse IgG antibody (KPL, Guilford, UK) with washing between each step 1% BSA/PBS-IFA. Finally, the cells were analyzed by flow cytometry on a BD FACSCalibur cytometer (Becton Dickinson, BD Biosciences, San Jose, CA) using the CELLQuest software. All experiments were performed independently in triplicate.

### Western blots

Mock and DENV 2 infected cells were treated with the appropriate concentration of VDR agonists for various periods of time (0, 1, 3, 6 and 12 hpi), after which the treated cells were collected and total protein was extracted using RIPA lysis buffer containing a protease inhibitor cocktail (Bio Basic Inc., Markham, Ontario, Canada). The protein concentration was determined by Bradford assay (Bio-rad, San Francisco, CA) and samples were stored at − 80 °C until used. Extracted protein was separated on 12% sodium dodecyl sulfate–polyacrylamide gel electrophoresis (SDS-PAGE) gels, followed by transfer to nitrocellulose membranes (Whatman GmbH, Germany). The membranes were subsequently blocked with 5% skim milk in 1X TBS/T (1X Tris-buffered saline containing 0.05% Tween-20) at room temperature and subsequently probed with a 1:500 dilution of a mouse monoclonal anti-dengue serotype 1–4 antibody (MA1-27093; Thermo Fisher Scientific Inc., Waltham, MA), a 1:2000 dilution of a rabbit polyclonal anti-dengue type 2 NS1 antibody (PA5-27885, Thermo Fisher Scientific Inc.), a 1:5,000 dilution of a rabbit polyclonal anti-dengue NS3 antibody (PA5-32199, Thermo Fisher Scientific Inc. Waltham, MA), a 1:3,000 dilution of a mouse monoclonal anti-dengue NS5 antibody (GTX629446, GeneTex, Irvine, CA) and a 1:5,000 dilution of a mouse monoclonal anti-GAPDH antibody (sc-32233, Santa Cruz, Dallas, TX) at 4 °C for overnight. Membranes were subsequently incubated with a 1:5,000 dilution of a horseradish peroxidase-conjugated polyclonal goat anti-rabbit IgG antibody (31460; Thermo Fisher Scientific Inc. Waltham, MA) or a 1:5,000 dilution of a horseradish peroxidase-conjugated polyclonal goat anti-mouse IgG antibody (31430; Thermo Fisher Scientific Inc. Waltham, MA). The signals were developed using the ECL Plus Western Blotting Analysis kit (Amersham Pharmacia Biotech, Piscataway, NJ), then the signal was detected using X-Ray film. Experiments were performed independently in triplicate.

### DENV RNA quantitation by real-time PCR

HEK293T/17 cells were mock-infected or infected with DENV 2 at MOI 5 for 2 h, followed by treated with 10 µM of VDR agonists at various time points. At 24 h.p.i., the supernatant of the treated cells was collected to determining DENV 2 genome copy number by quantitative real-time PCR (qPCR) using the KAPA SYBR FAST qPCR Kit 2X Master MIX (Kapa Biosystems Inc., Woburn, MA.). Total RNA was extracted using Trizol reagent (Thermo Fisher Scientific Inc., Waltham, MA), then cDNA was synthesized using random hexamer primers and RevertAid reverse transcriptase (Thermo Fisher Scientific Inc., Waltham, MA). Amplification was performed using specific primers to the DENV NS1 portion of the viral genome, namely NS1-F: 5′-CAATATGCTGAAACGCGAGAGAAA-3′ and NS1-R: 5′-CCCCATCTATTCAGAATCCCTGCT-3′. DENV copy number was calculated from the cycle threshold value of the amplification plot, compared with the tenfold serial dilution of a DENV genome standard control. Experiments were performed independently in triplicate with duplicate real-time PCR.

### VDR-regulated gene expression by quantitative real-time PCR

HEK293T/17 cells were incubated with either 10 µM of VDR agonists or 20 μM of commercial VDR agonist EB1089 (Tocris Cookson Ltd., Bristol, UK) for 24 h. Subsequently, the treated cell lysate was collected to determine the mRNA levels of the VDR gene as well as genes regulated by VDR. Total RNA was extracted and cDNA was synthesized as described above. Amplification was performed using specific primers, namely VDR-F: 5′-TGCTATGACCTGTGAAGGCTG-3′, VDR-R: 5′-AGTGGCGTCGGTTGTCCTT-3′, CYP27B1-F: 5′-GAATTGCAAATGGCTTTGGCCCAG-3′, CYP27B1-R 5′-CTGTAGGTTGATGCTCCTTTCAGG-3′, CYP24A1-F: 5′-CAGCGAACTGAACAAATGGTCG-3′ and CYP24A1-R 5′-TCTCTTCTCATACAACACGAGGCAG-3′. The mRNA expression was normalized against β-actin and DMSO control-treated cells, respectively according to the following equation: ΔΔCt = ΔCt (gene)—ΔCt (DMSO). Experiments were performed independently in triplicate with triplicate real-time PCR.

### Immunofluorescence assay

HEK293T/17 cells grown on glass coverslips were mock-infected or infected with DENV 2 followed by treatment with 10 µM of VDR agonists for 24 h, after which time cells were fixed with 4% ice-cold paraformaldehyde. Then the cells were washed twice with 1X PBS/IFA and blocked with 10% goat serum. After washing twice with 0.03% Triton X-100 in PBS/IFA, the cells were permeabilized with 0.3% Triton X-100 in PBS. The cells were incubated with a mouse monoclonal anti-dengue serotype 1–4 antibody (MA1-27093; Thermo Fisher Scientific Inc., MA) and a rabbit monoclonal anti-VDR antibody (Abcam: ab3508), both at a dilution of 1:200. Cells were subsequently incubated with an Alexa 488-conjugated donkey anti-mouse IgG antibody (A21202, Thermo Fisher Scientific Inc., Waltham, MA) and an Alexa 647-conjugated donkey anti-rabbit IgG antibody (A31573, Thermo Fisher Scientific Inc., Waltham, MA), together with a 1:500 dilution of DAPI for 1 h at room temperature. The coverslips were subsequently mounted onto glass slides using Prolong Gold anti-fade reagent (Invitrogen) before visualization under a LSM 800w Airy scan confocal microscope (ZEISS, Oberkochen, Germany)**.**

### Statistical analysis

All data were analyzed using the GraphPad Prism program (GraphPad Software Inc., San Diego, CA). Statistical significance was evaluated using the independent t-test of PASW Statistics 18.0.0 (SPSS Inc., Chicago, IL) with p-values of *< 0.05, **< 0.01, ***< 0.001 for significance. EC_50_ and CC_50_ values were calculated using the freeware ED50plus (v1.0) software (https://sciencegateway.org/protocols/cellbio/drug/data/ed50v10.xls).

## Data availability statement

All data generated or analysed during this study are included in this published article (and its Supplementary Information files).

## Supplementary information

Supplementary file1

Supplementary file2

Supplementary file3

Supplementary file4

## References

[1] Bhatt S (2013). The global distribution and burden of dengue. Nature.

[2] Guzman MG, Harris E (2015). Dengue. Lancet.

[3] Gubler DJ (1998). Dengue and dengue hemorrhagic fever. Clin. Microbiol. Rev..

[4] Fatima K, Syed NI (2018). Dengvaxia controversy: Impact on vaccine hesitancy. J. Glob. Health.

[5] Halstead SB (2017). Dengvaxia sensitizes seronegatives to vaccine enhanced disease regardless of age. Vaccine.

[6] Chang J, Block TM, Guo J-T (2013). Antiviral therapies targeting host ER alpha-glucosidases: Current status and future directions. Antiviral Res..

[7] Courageot M-P, Frenkiel M-P, Dos Santos CD, Deubel V, Desprès P (2000). α-Glucosidase inhibitors reduce dengue virus production by affecting the initial steps of virion morphogenesis in the endoplasmic reticulum. J. Virol..

[8] Holick MF (2007). Vitamin D deficiency. N. Engl. J. Med..

[9] Baeke F, Takiishi T, Korf H, Gysemans C, Mathieu C (2010). Vitamin D: Modulator of the immune system. Curr. Opin. Pharmacol..

[10] Nagpal S, Na S, Rathnachalam R (2005). Noncalcemic actions of vitamin D receptor ligands. Endocr. Rev..

[11] Haussler MR (2013). Molecular mechanisms of vitamin D action. Calcif. Tissue Int..

[12] Kongsbak M, Levring T, Geisler C, von Essen M (2013). The vitamin D receptor and T cell function. Front. Immunol..

[13] Alagarasu K (2017). In-vitro effect of human cathelicidin antimicrobial peptide LL-37 on dengue virus type 2. Peptides.

[14] Aranow C (2011). Vitamin D and the immune system. J. Invest. Med..

[15] Gal-Tanamy M (2011). Vitamin D: an innate antiviral agent suppressing hepatitis C virus in human hepatocytes. Hepatology.

[16] Alvarez N, Aguilar-Jimenez W, Rugeles MT (2019). The potential protective role of vitamin D supplementation on HIV-1 infection. Front. Immunol..

[17] Gupta S (2019). The role of micronutrients in the infection and subsequent response to hepatitis C virus. Cells.

[18] Alagarasu K, Bachal RV, Bhagat AB, Shah PS, Dayaraj C (2012). Elevated levels of vitamin D and deficiency of mannose binding lectin in dengue hemorrhagic fever. Virol. J..

[19] Alagarasu K (2012). Association of vitamin D receptor gene polymorphisms with clinical outcomes of dengue virus infection. Hum. Immunol..

[20] Puerta-Guardo H, Hernández SI, Rosales VH, Ludert JE, del Angel RM (2012). The 1α,25-dihydroxy-vitamin D3 reduces dengue virus infection in human myelomonocyte (U937) and hepatic (Huh-7) cell lines and cytokine production in the infected monocytes. Antiviral Res..

[21] Arboleda Alzate JF, Rodenhuis-Zybert IA, Hernandez JC, Smit JM, Urcuqui-Inchima S (2017). Human macrophages differentiated in the presence of vitamin D3 restrict dengue virus infection and innate responses by downregulating mannose receptor expression. PLoS Negl. Trop. Dis..

[22] Xu B (2019). Discovery of fused bicyclic derivatives of 1H-pyrrolo[1,2-c]imidazol-1-one as VDR signaling regulators. Bioorg Med. Chem..

[23] Pike JW, Meyer MB (2010). The vitamin D receptor: New paradigms for the regulation of gene expression by 1,25-dihydroxyvitamin D(3). Endocrinol. Metab. Clin. N. Am..

[24] Bitetto D (2011). Vitamin D supplementation improves response to antiviral treatment for recurrent hepatitis C. Transpl. Int..

[25] Ravid A (2019). 25-Hydroxyvitamin D inhibits hepatitis C virus production in hepatocellular carcinoma cell line by a vitamin D receptor-independent mechanism. Int. J. Mol. Sci..

[26] Giraldo DM, Cardona A, Urcuqui-Inchima S (2018). High-dose of vitamin D supplement is associated with reduced susceptibility of monocyte-derived macrophages to dengue virus infection and pro-inflammatory cytokine production: An exploratory study. Clin. Chim. Acta.

[27] Jadhav NJ, Gokhale S, Seervi M, Patil PS, Alagarasu K (2018). Immunomodulatory effect of 1, 25 dihydroxy vitamin D3 on the expression of RNA sensing pattern recognition receptor genes and cytokine response in dengue virus infected U937-DC-SIGN cells and THP-1 macrophages. Int. Immunopharmacol..

[28] Nurminen V, Seuter S, Carlberg C (2019). Primary vitamin D target genes of human monocytes. Front. Physiol..

[29] Heaton NS, Randall G (2010). Dengue virus-induced autophagy regulates lipid metabolism. Cell Host Microbe.

[30] Lee YR (2008). Autophagic machinery activated by dengue virus enhances virus replication. Virology.

[31] Panyasrivanit M, Khakpoor A, Wikan N, Smith DR (2009). Co-localization of constituents of the dengue virus translation and replication machinery with amphisomes. J. Gen. Virol..

[32] Haas MJ, Jafri M, Wehmeier KR, Onstead-Haas LM, Mooradian AD (2016). Inhibition of endoplasmic reticulum stress and oxidative stress by vitamin D in endothelial cells. Free Radic. Biol. Med..

[33] Datan E (2016). Dengue-induced autophagy, virus replication and protection from cell death require ER stress (PERK) pathway activation. Cell Death Dis..

[34] Klomporn P, Panyasrivanit M, Wikan N, Smith DR (2011). Dengue infection of monocytic cells activates ER stress pathways, but apoptosis is induced through both extrinsic and intrinsic pathways. Virology.

[35] Lee YR (2018). Dengue virus-induced ER stress is required for autophagy activation, viral replication, and pathogenesis both in vitro and in vivo. Sci Rep.

[36] Chen HH (2017). AR-12 suppresses dengue virus replication by down-regulation of PI3K/AKT and GRP78. Antiviral Res..

[37] Thepparit C (2013). Dengue 2 infection of HepG2 liver cells results in endoplasmic reticulum stress and induction of multiple pathways of cell death. BMC Res. Notes.

[38] Wati S (2009). Dengue virus infection induces upregulation of GRP78, which acts to chaperone viral antigen production. J. Virol..

[39] Pear WS, Nolan GP, Scott ML, Baltimore D (1993). Production of high-titer helper-free retroviruses by transient transfection. Proc. Natl. Acad. Sci. USA.

[40] Holick MF (2017). The vitamin D deficiency pandemic: Approaches for diagnosis, treatment and prevention. Rev. Endocr. Metab. Disord..

[41] Villamor E, Villar LA, Lozano A, Herrera VM, Herran OF (2017). Vitamin D serostatus and dengue fever progression to dengue hemorrhagic fever/dengue shock syndrome. Epidemiol. Infect..

[42] Sanchez-Valdez E, Delgado-Aradillas M, Torres-Martinez JA, Torres-Benitez JM (2009). Clinical response in patients with dengue fever to oral calcium plus vitamin D administration: Study of 5 cases. Proc. West. Pharmacol. Soc..

[43] Sithisarn P, Suksanpaisan L, Thepparit C, Smith DR (2003). Behavior of the dengue virus in solution. J. Med. Virol..

[44] Henchal EA, Gentry MK, McCown JM, Brandt WE (1982). Dengue virus-specific and flavivirus group determinants identified with monoclonal antibodies by indirect immunofluorescence. Am. J. Trop. Med. Hyg..

