# Supplementary material for: Activity of vitamin D receptor agonists against dengue virus
Source: Sci Rep. 2020 Jul 2;10:10835. doi: 10.1038/s41598-020-67783-z (PMC7331731; doi:10.1038/s41598-020-67783-z)
Supplement: Supplementary file 1 — Supplementary file1 [file 41598_2020_67783_MOESM1_ESM.pdf]

Supplemental Materials

Activity of vitamin D receptor agonists against dengue virus

Janejira Jaratsittisin[1], Bin Xu[2], Wannapa Sornjai[1], Zhibing Weng[2], Atichat Kuadkitkan[1], Feng Li[2], Guo-Chun Zhou[2*], Duncan R. Smith[1*]

[1]Institute of Molecular Biosciences, Mahidol University, Salaya, 73170, Thailand

[2] School of Pharmaceutical Sciences, Nanjing Tech University, Nanjing 211816, Jiangsu, China

[*]Correspondence should be addressed to gczhou@njtech.edu.cn (GCZ) or duncan_r_smith@hotmail.com (DRS)

Supplemental Figure S1. Cytotoxicity of vitamin D receptor agonists by trypan blue staining and MTT assay

Supplemental Figure S2. Cytotoxicity of vitamin D receptor agonists by the alteration of cell morphology

Supplemental Figure S3. Half-maximum effective concentration (EC_{50}) of vitamin D receptor agonists

Supplemental Figure S4. Virucidal activity of vitamin D receptor agonists

Supplementary Table S1. Chemical structures of VDR agonists

Legend to Supplemental movies. VDR localization by immunofluorescence assay

Uncropped western blots

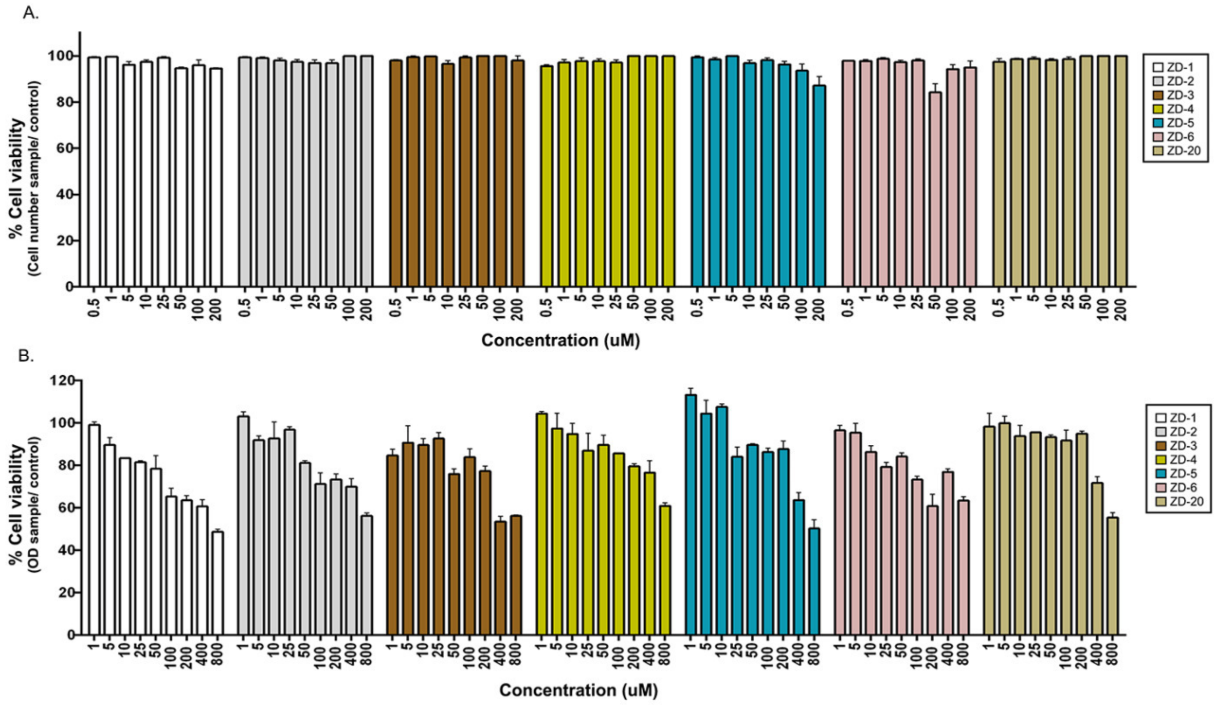

Supplemental Figure S1. Cytotoxicity of vitamin D receptor agonists by trypan blue staining and MTT assay. HEK293T/17 cells were cultured and incubated with the indicated various concentrations of VDR agonists for 24h. the treated cells were examined the cytotoxicity using 0.4% trypan blue staining (A) and MTT assay (B). The percentage of cell viability was calculated compared with complete media treated cells. The experiments were performed independently triplicate for trypan blue staining and four replicates for MTT assay.

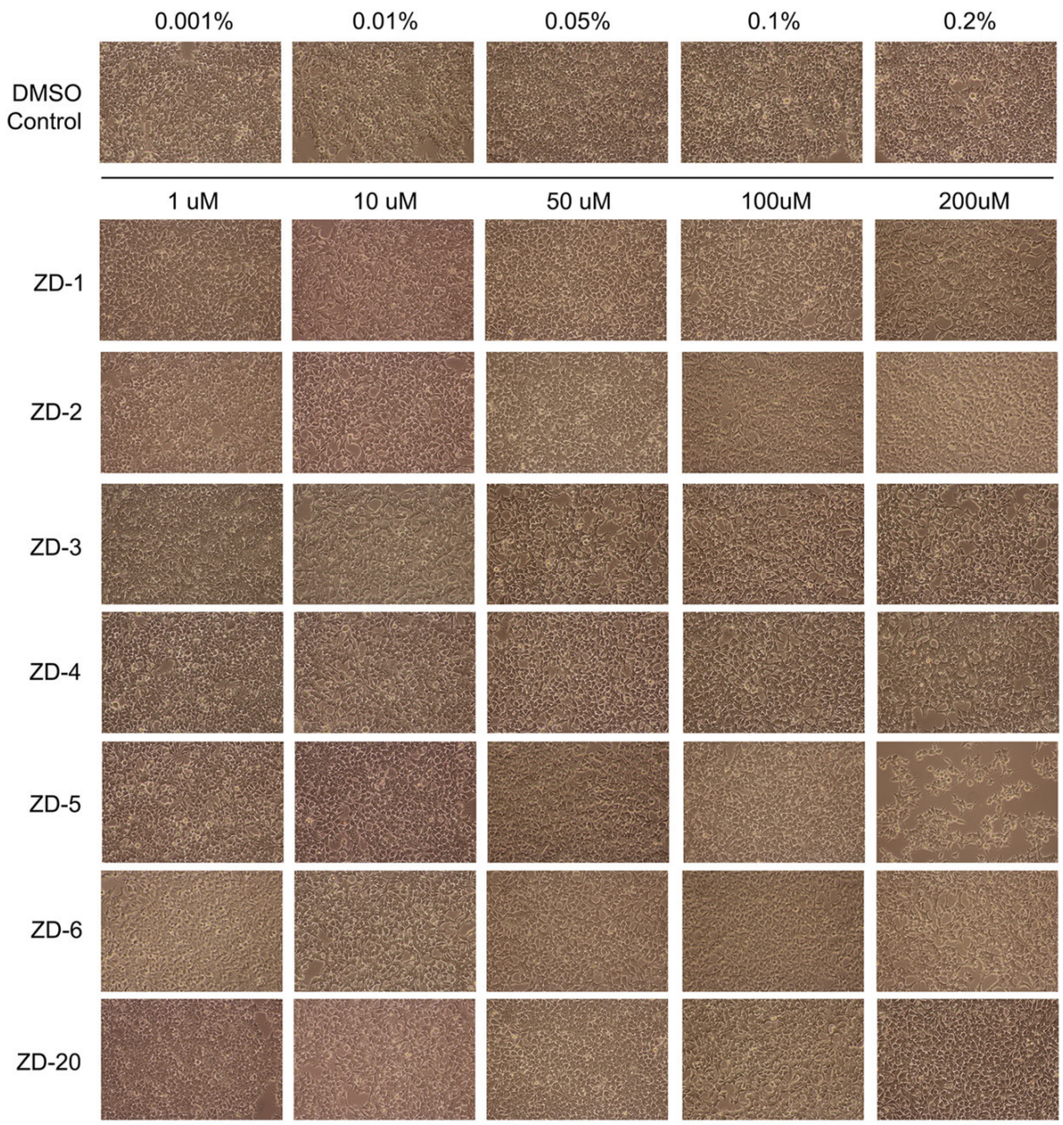

Supplemental Figure S2. Cytotoxicity of vitamin D receptor agonists by the alteration of cell morphology. HEK293T/17 cells were cultured and treated with various concentrations of VDR agonists (ZD-1, ZD-2, ZD-3, ZD-4, ZD-5, ZD-6 and ZD-20), together with the DMSO diluent control. After the incubation for 24h, the treated cells were observed and captured using inverted microscopy. The experiments were performed independently triplicate.

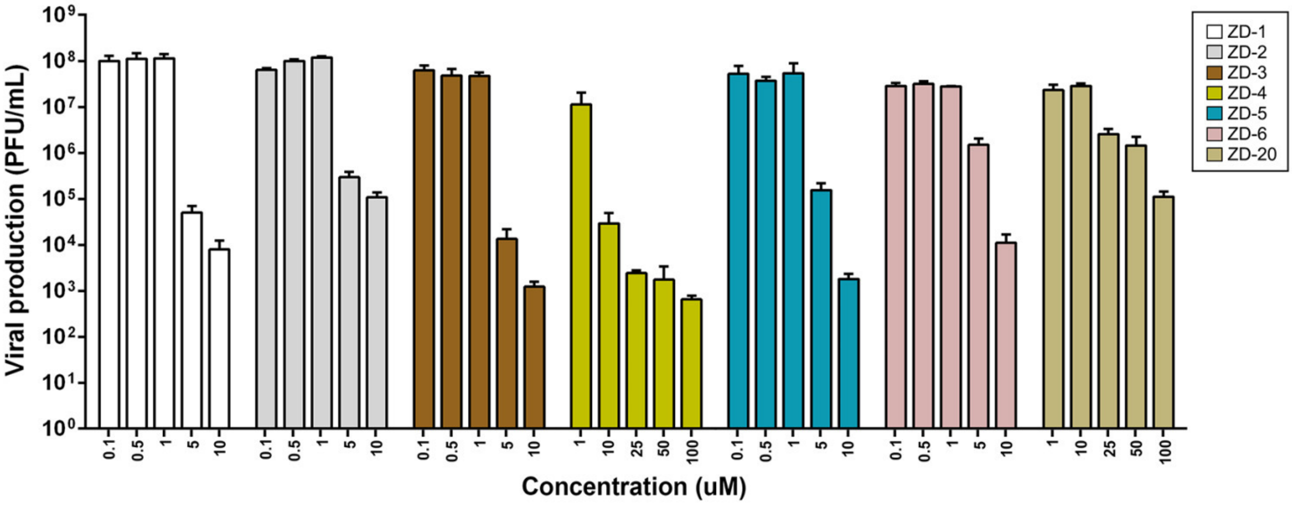

Supplemental Figure S3. Half-maximum effective concentration (EC_{50}) of vitamin D receptor agonists. HEK293T/17 cells were incubated with 0.1-10 μM of ZD-1, -2, -3, -5, -6 or 1-100 μM of ZD-4 and -20 for 24 h. The supernatant was collected and viral production determined via standard plaque assay. The experiments were performed independently in triplicate with duplicate plaque assay.

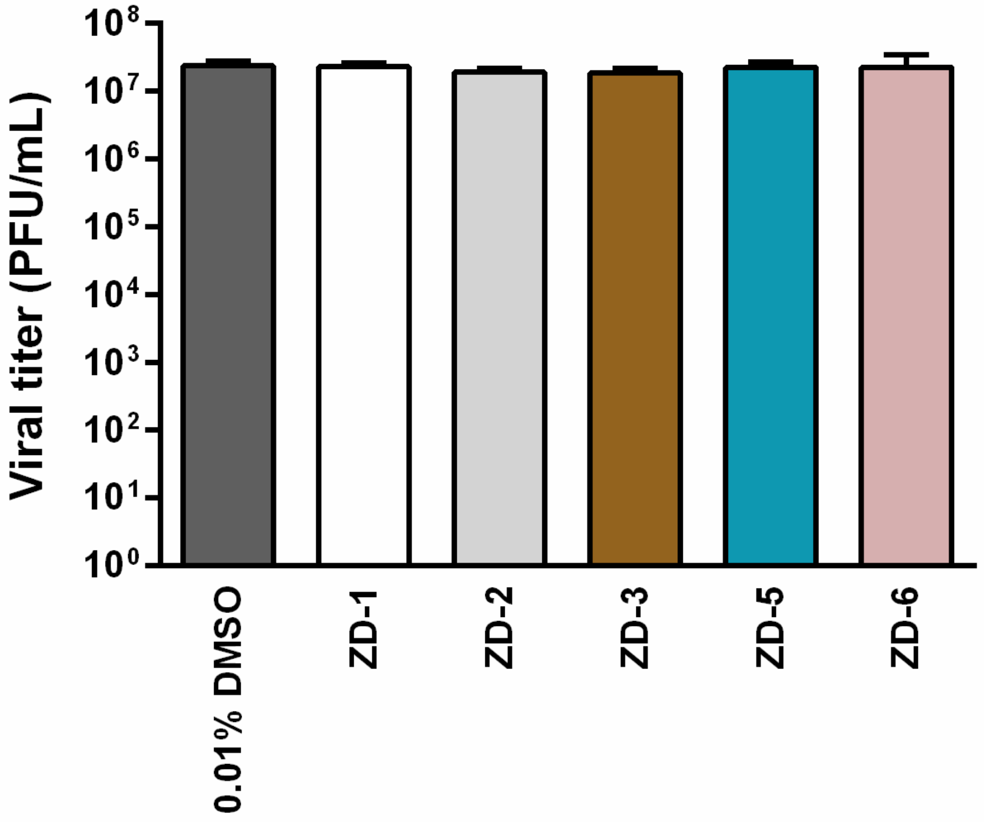

Supplemental Figure S4. Virucidal activity of vitamin D receptor agonists. A mixture of DENV 2 and 10 μM of vitamin D receptor agonists were incubated at 37°C for one hour before determining virus titer by standard plaque assay. DMSO treated virus was used as a control of this experiment. The experiment was performed independently in triplicate and duplicate in plaque assay.

Supplementary Table S1. Structures, IDs, compound ID, chemical formulas and formula weights of 7 VDR agonists.

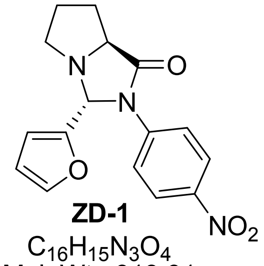 **ZD-1** $C_{16}H_{15}N_3O_4$ Mol. Wt.: 313.31	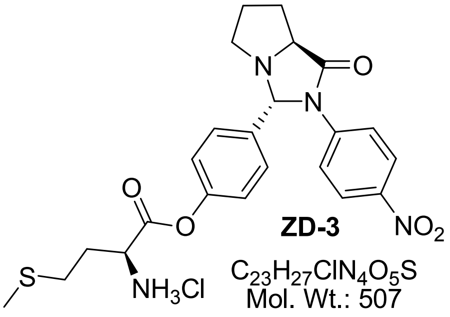 **ZD-3** $C_{23}H_{27}ClN_4O_5S$ Mol. Wt.: 507	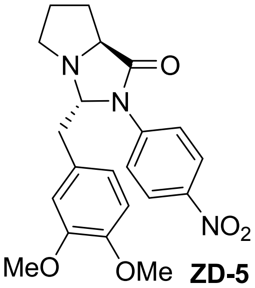 **ZD-5** $C_{21}H_{23}N_3O_5$ Mol. Wt.: 397.42
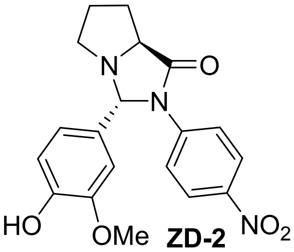 **ZD-2** $C_{19}H_{19}N_3O_5$ Mol. Wt.: 369.37	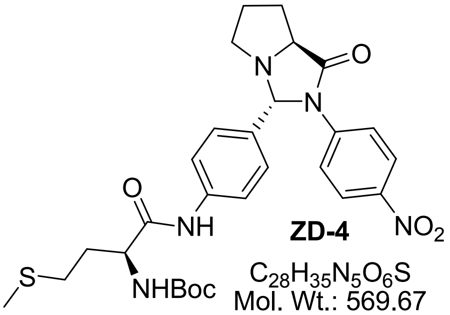 **ZD-4** $C_{28}H_{35}N_5O_6S$ Mol. Wt.: 569.67	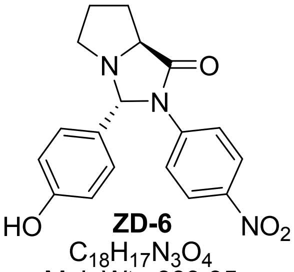 **ZD-6** $C_{18}H_{17}N_3O_4$ Mol. Wt.: 339.35
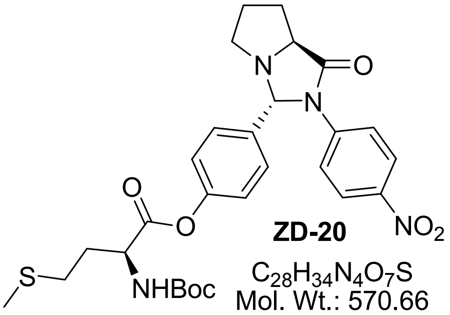 **ZD-20** $C_{28}H_{34}N_4O_7S$ Mol. Wt.: 570.66		

Supplemental movie 1. VDR localization by immunofluorescence assay. HEK293T/17 cells were (A) mock-infected, (B) DENV 2 infected or (C) DENV 2 infected and treated with 10 μM ZD-6. After 24h of treatment, cells were processed under standard protocol of immunofluorescence assay. DENV E (green) protein and VDR (red) were detected using specific antibodies. Cells were stained with DAPI (blue). All signals were observed and produced as a 3D structure movie under confocal microscope LSM 800 w Airyscan (ZEISS, Oberkochen , Germany).

Time-of-additional effect of VDR agonist
on viral protein expression via western blot assay
VDR agonists: ZD-1, ZD-2, ZD-3, ZD-5 and ZD-6
(Protein: NS5, NS3, E, NS1 and GAPDH)

ZD-1

ZD-1: NS5

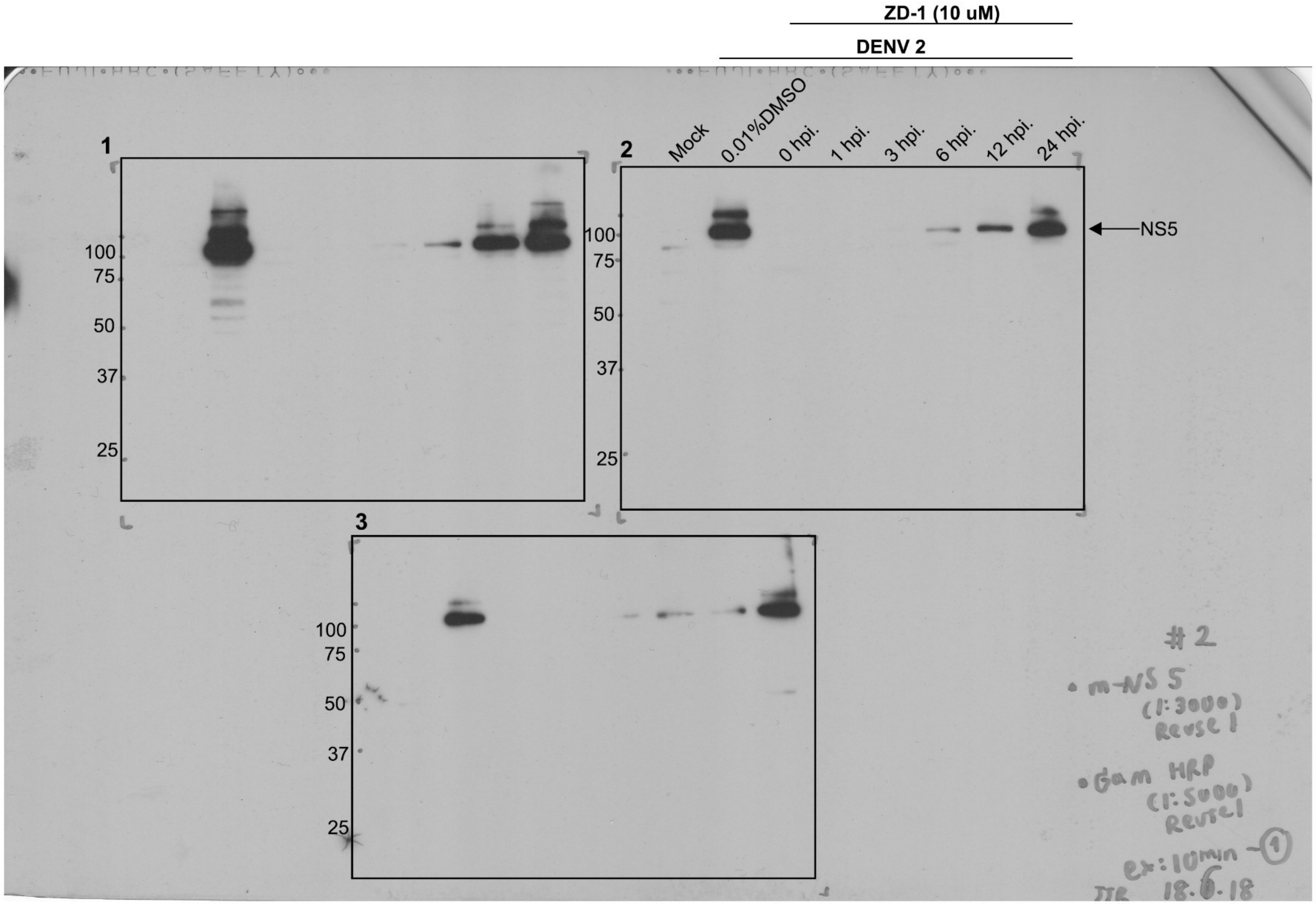
ZD-1 (10 uM)
DENV 2
Mock
0.01%DMSO
0 hpi.
1 hpi.
3 hpi.
6 hpi.
12 hpi.
24 hpi.
1
2
3
100
75
50
37
25
NS5
#2
• m-NS5 (1:3000) Revsel
• Gam HRP (1:5000) Revsel
ex: 10min
18.6.18

ZD-1: NS3

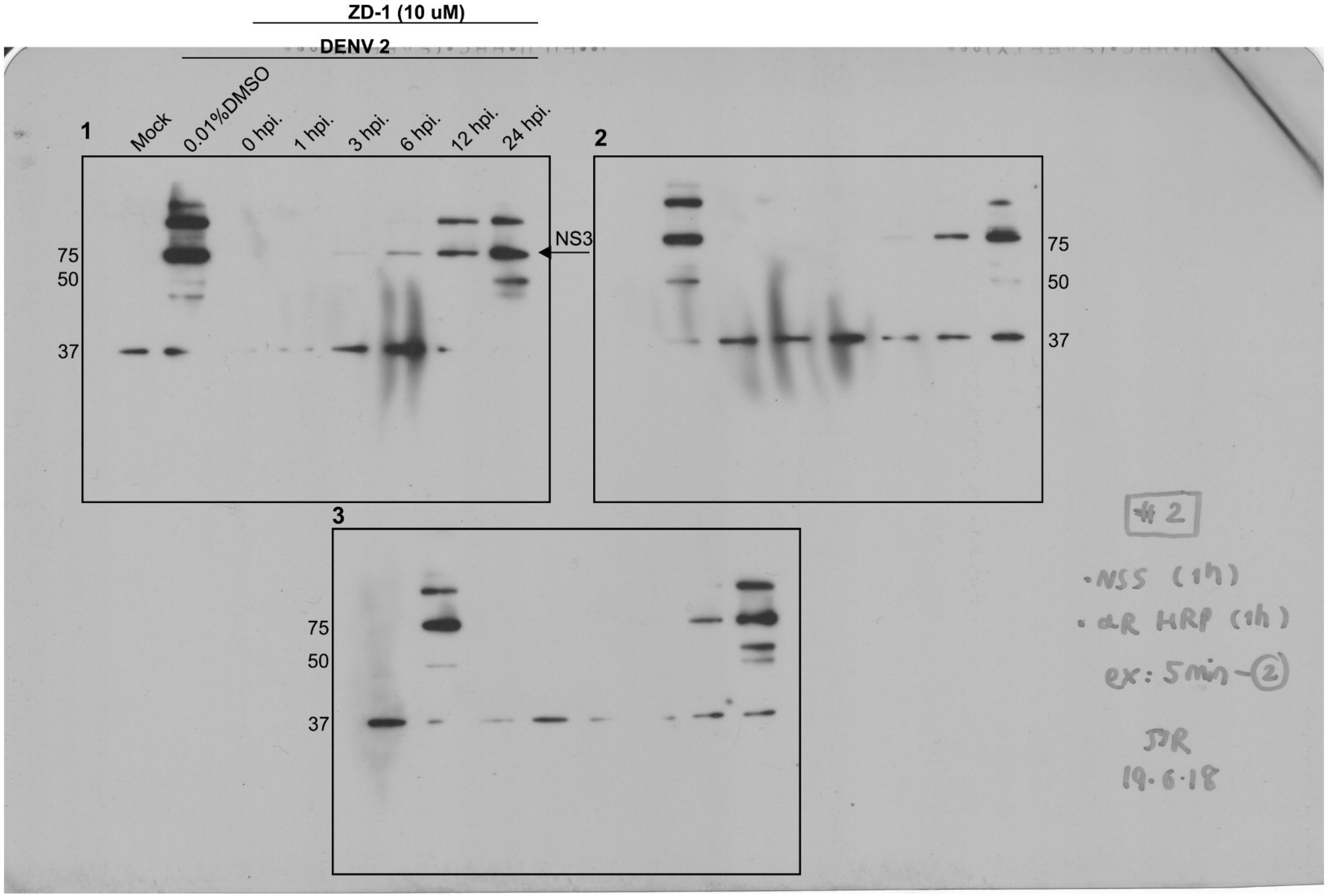
ZD-1 (10 uM)
DENV 2
1
Mock
0.01%DMSO
0 hpi.
1 hpi.
3 hpi.
6 hpi.
12 hpi.
24 hpi.
2
NS3
75
50
37
75
50
37
3
75
50
37
#2
·NS5 (1h)
· αR HRP (1h)
ex: 5min - 2
JJR
19.6.18

ZD-1: E

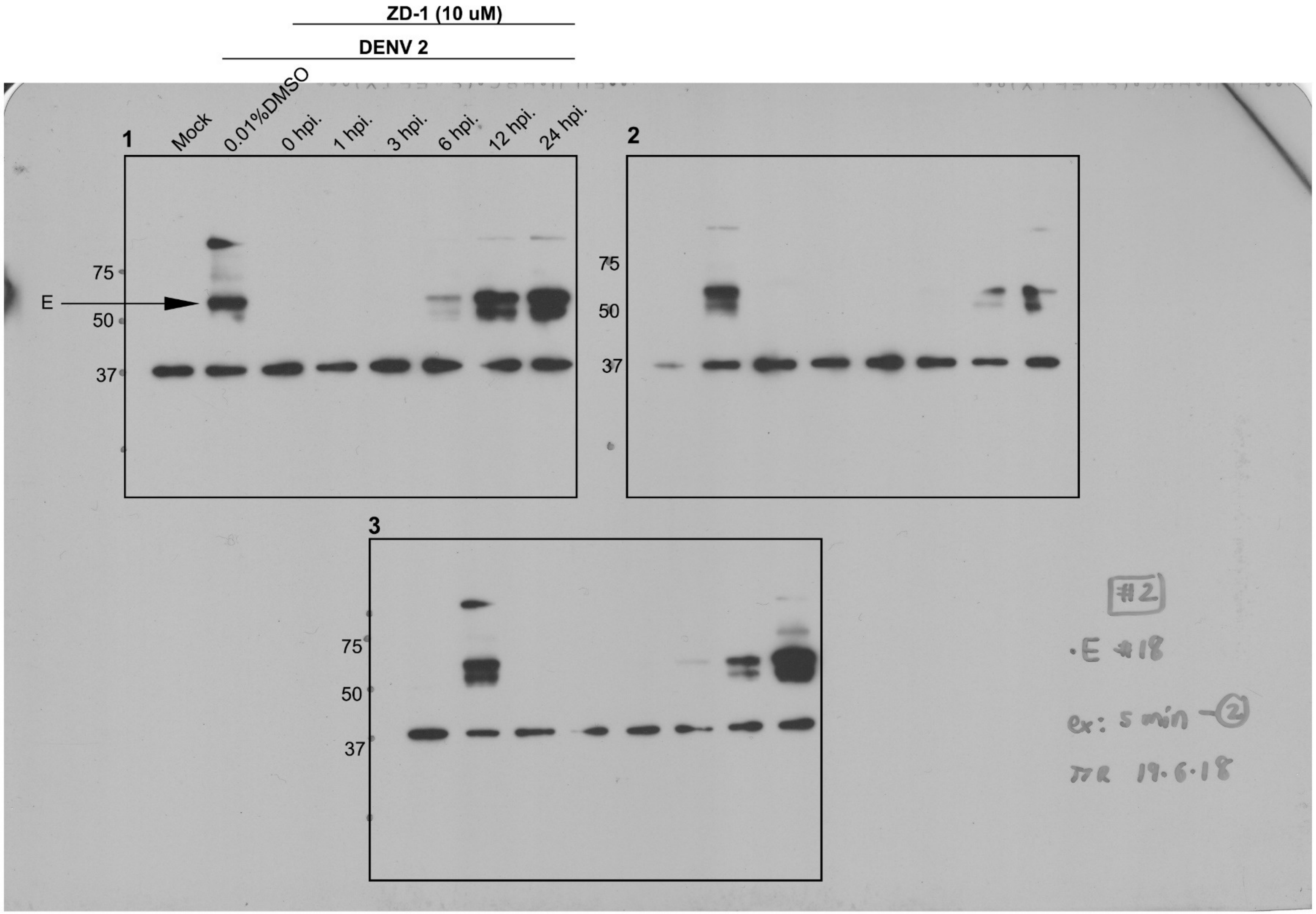
ZD-1 (10 uM)
DENV 2
1
Mock
0.01%DMSO
0 hpi.
1 hpi.
3 hpi.
6 hpi.
12 hpi.
24 hpi.
75
E
50
37
2
75
50
37
3
75
50
37
#2
·E #18
ex: 5 min -②
JJR 19.6.18

ZD-1: NS1

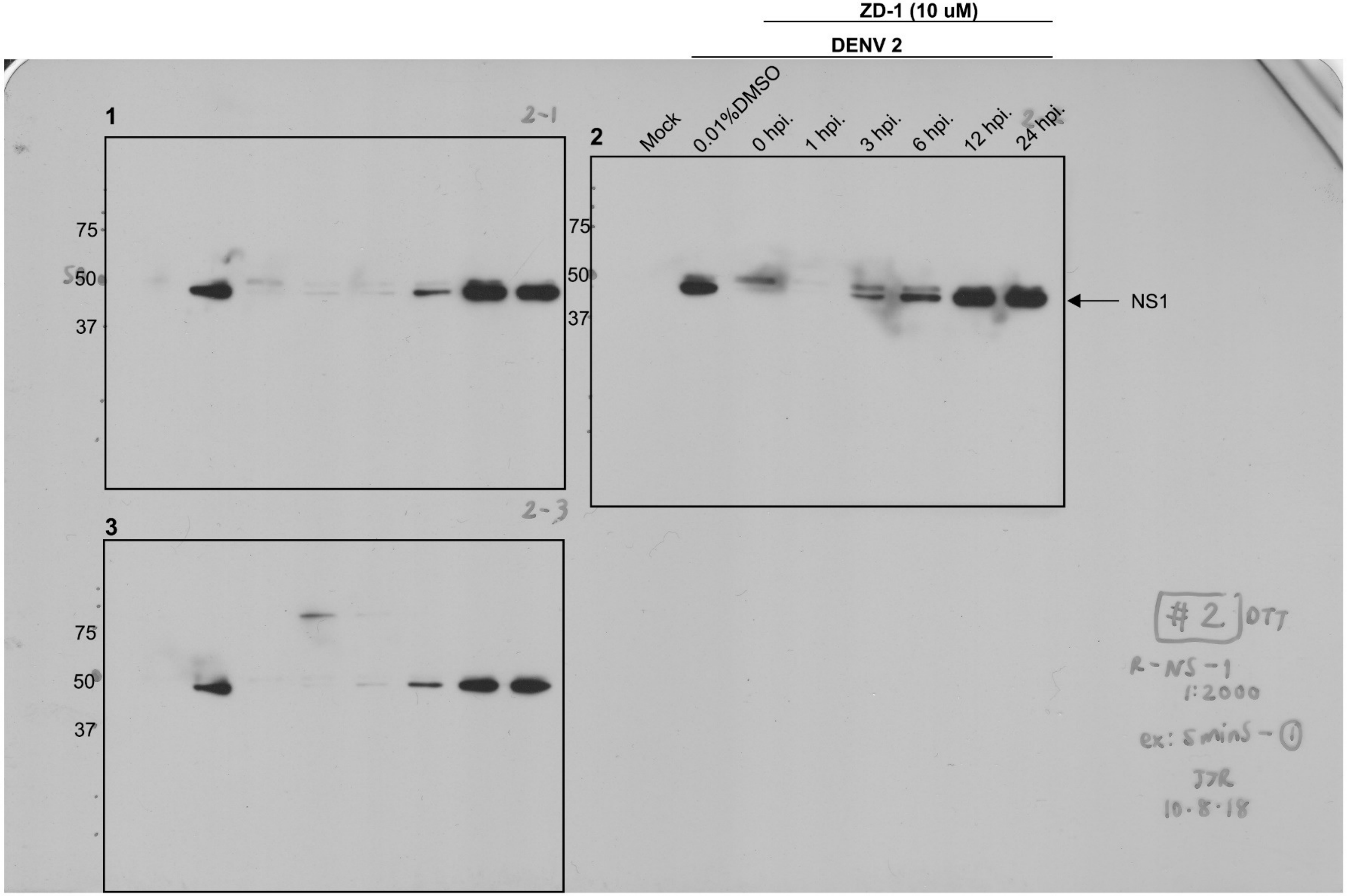
ZD-1 (10 uM)
DENV 2
1
2-1
2
Mock
0.01%DMSO
0 hpi.
1 hpi.
3 hpi.
6 hpi.
12 hpi.
24 hpi.
75
50
37
NS1
3
2-3
#2 DTT
R-NS-1
1:2000
ex: 5mins - 1
JJR
10.8.18

ZD-1: GAPDH

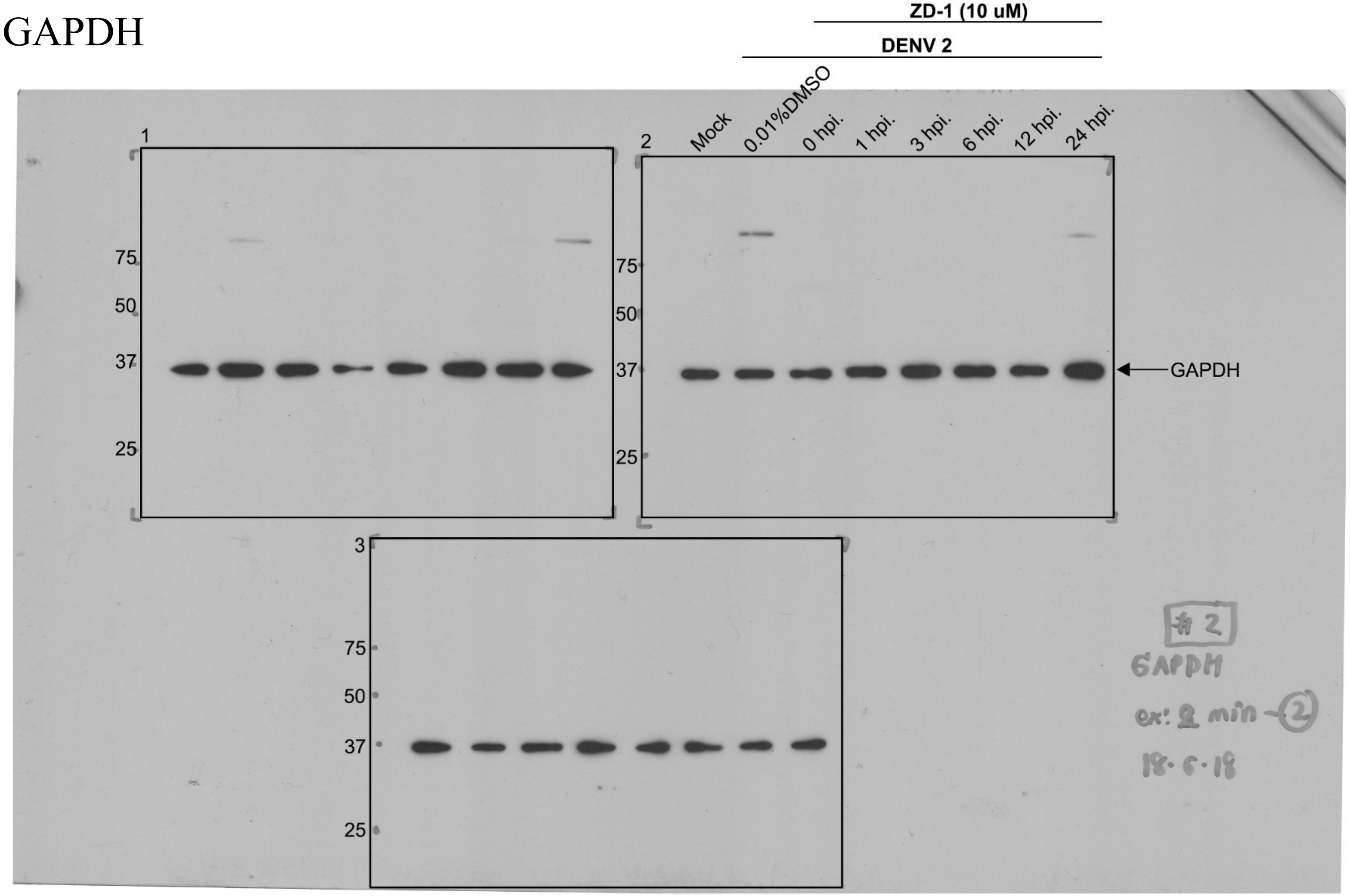

ZD-1 (10 uM)
DENV 2
Mock
0.01%DMSO
0 hpi.
1 hpi.
3 hpi.
6 hpi.
12 hpi.
24 hpi.
1
2
3
75
50
37
25
GAPDH
#2
GAPDH
ex: 2 min - 2
18.6.18

ZD-2

ZD-2: NS5

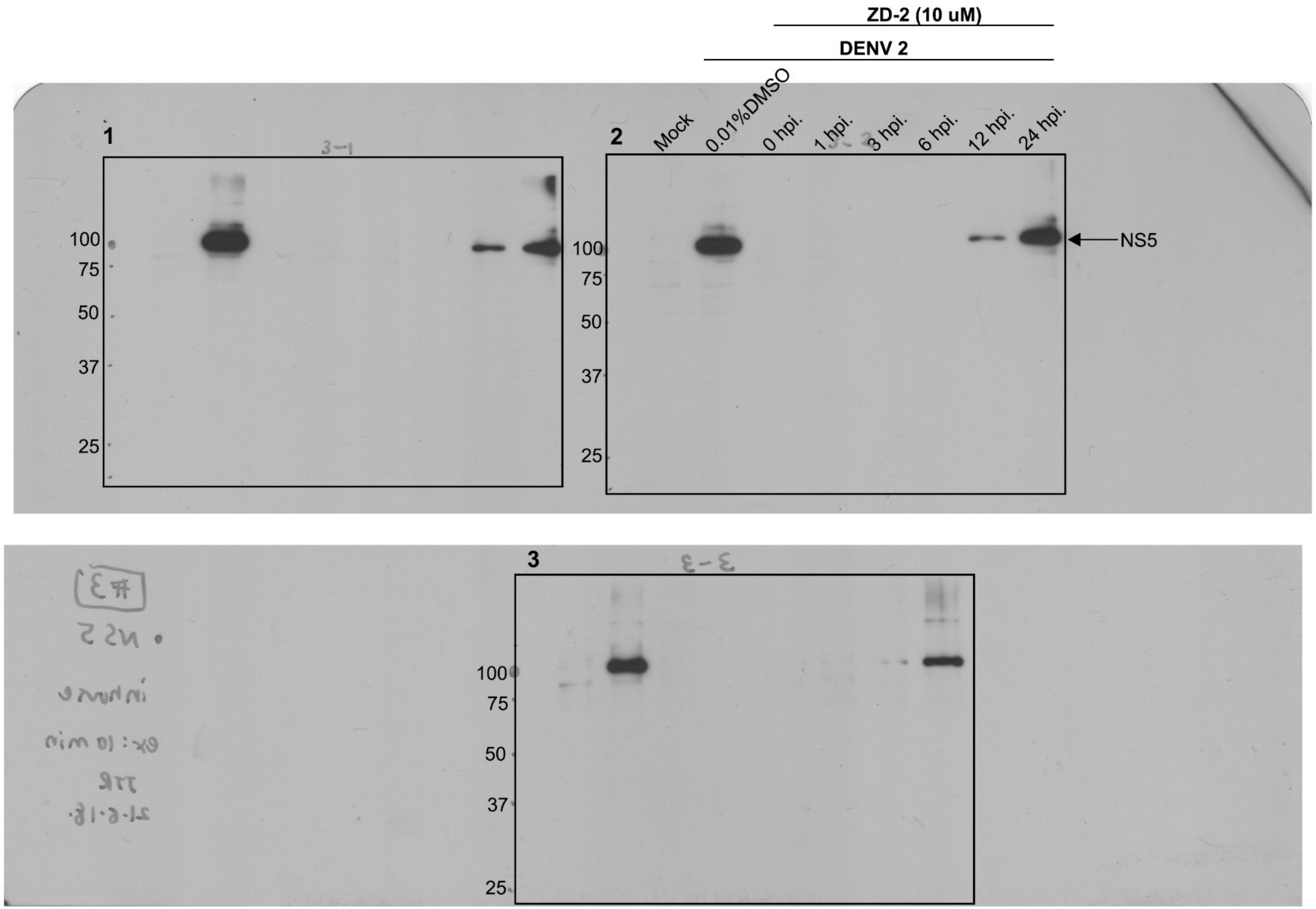

ZD-2 (10 uM)
DENV 2
1
2
Mock
0.01%DMSO
0 hpi.
1 hpi.
3 hpi.
6 hpi.
12 hpi.
24 hpi.
NS5
100
75
50
37
25
3

ZD-2: NS3

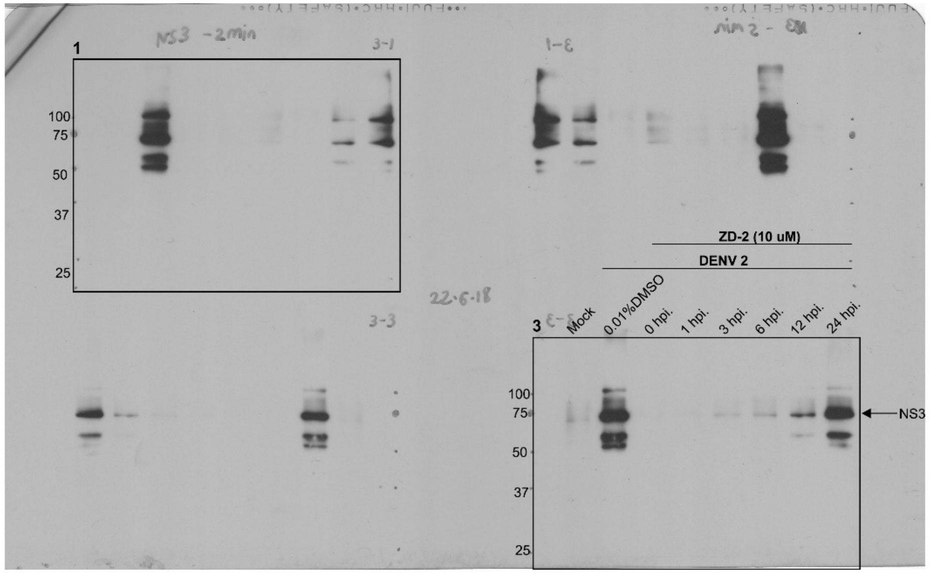

1
3
100
75
50
37
25
ZD-2 (10 uM)
DENV 2
Mock
0.01%DMSO
0 hpi.
1 hpi.
3 hpi.
6 hpi.
12 hpi.
24 hpi.
NS3

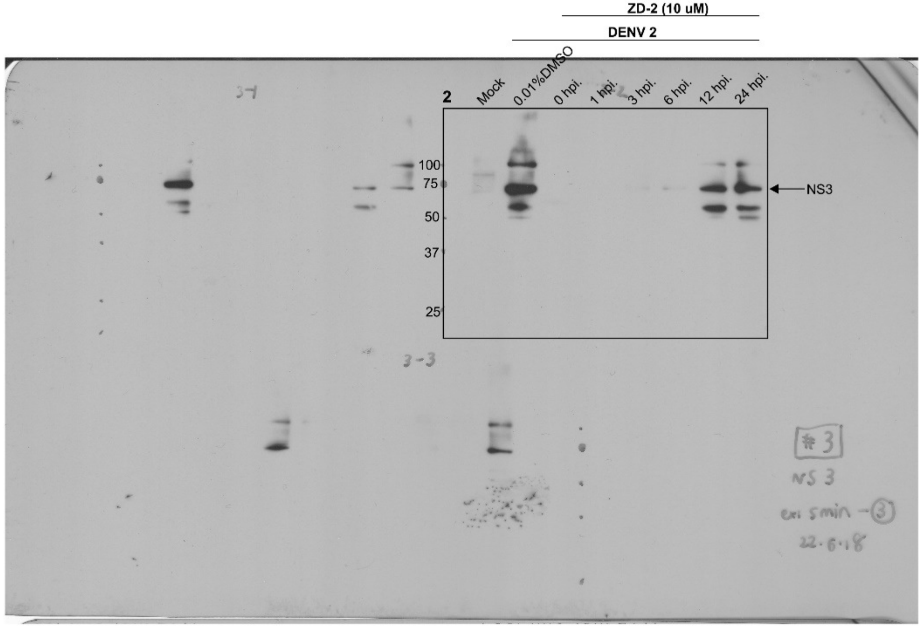

ZD-2 (10 uM)
DENV 2
2
Mock
0.01%DMSO
0 hpi.
1 hpi.
3 hpi.
6 hpi.
12 hpi.
24 hpi.
100
75
50
37
25
NS3

ZD-2: E

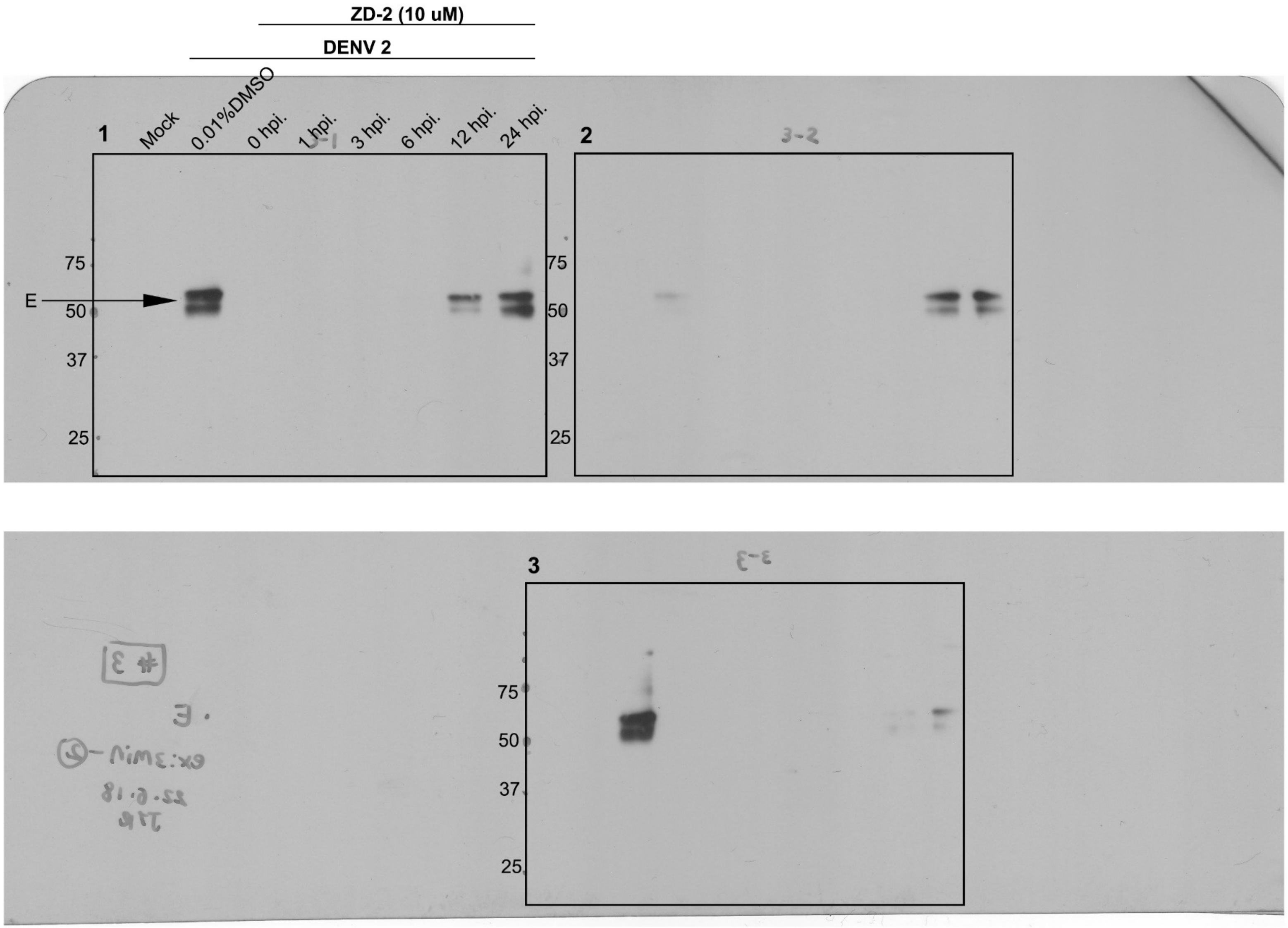
ZD-2 (10 uM)
DENV 2
1
Mock
0.01%DMSO
0 hpi.
1 hpi.
3 hpi.
6 hpi.
12 hpi.
24 hpi.
2
75
E
50
37
25
3
75
50
37
25

ZD-2: NS1

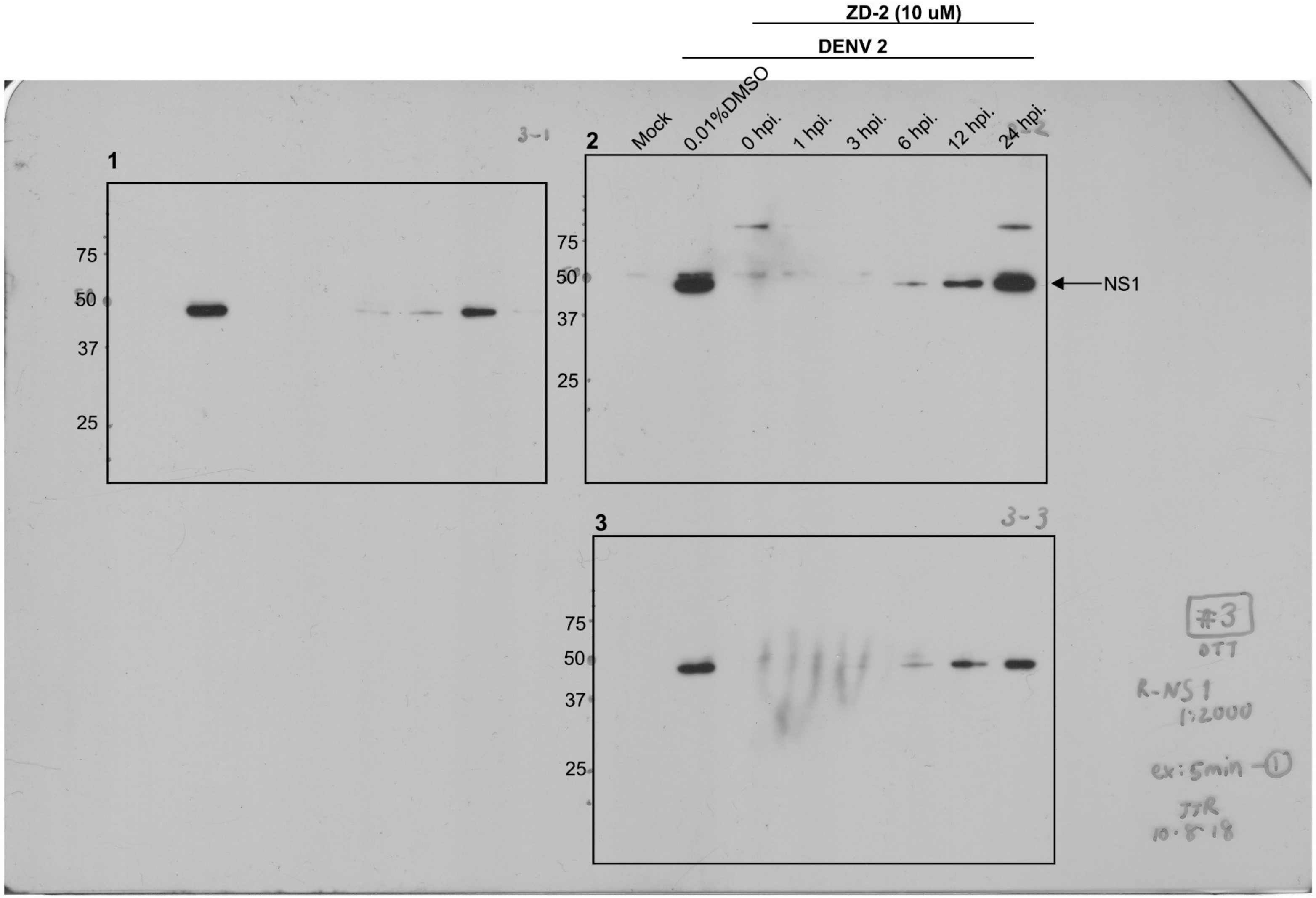

ZD-2 (10 uM)
DENV 2
Mock
0.01%DMSO
0 hpi.
1 hpi.
3 hpi.
6 hpi.
12 hpi.
24 hpi.
1
2
3
75
50
37
25
NS1
3-1
3-3
#3
DTT
R-NS1
1:2000
ex: 5min -①
JJR
10.8.18

ZD-2: GAPDH

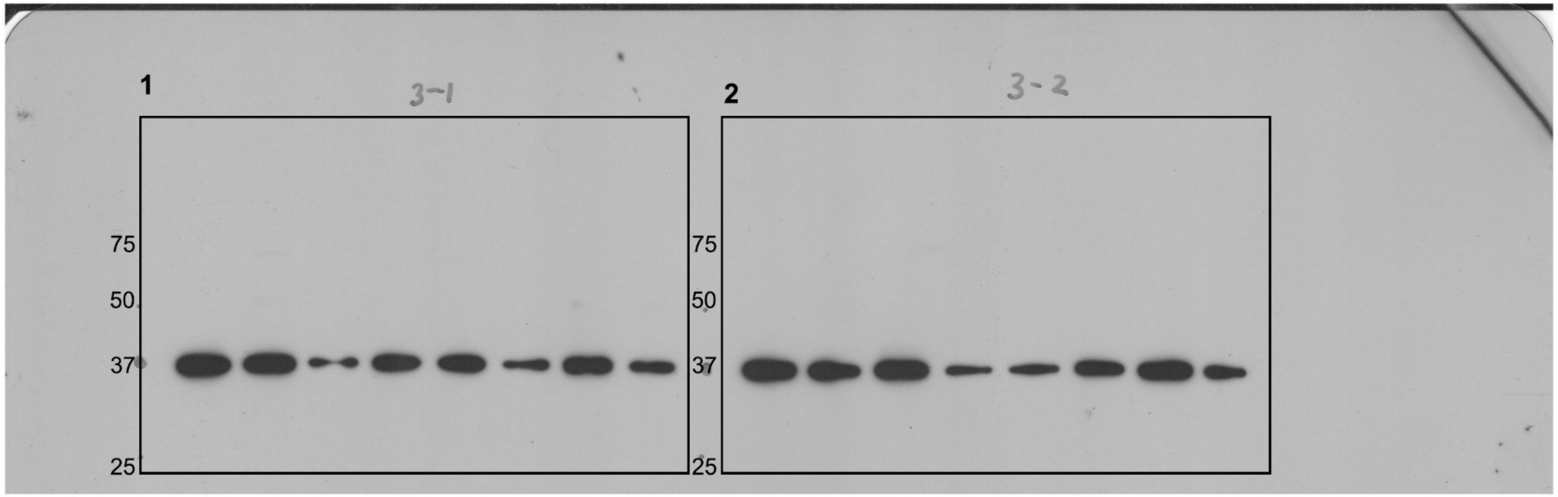
1
3-1
2
3-2
75
50
37
25
75
50
37
25

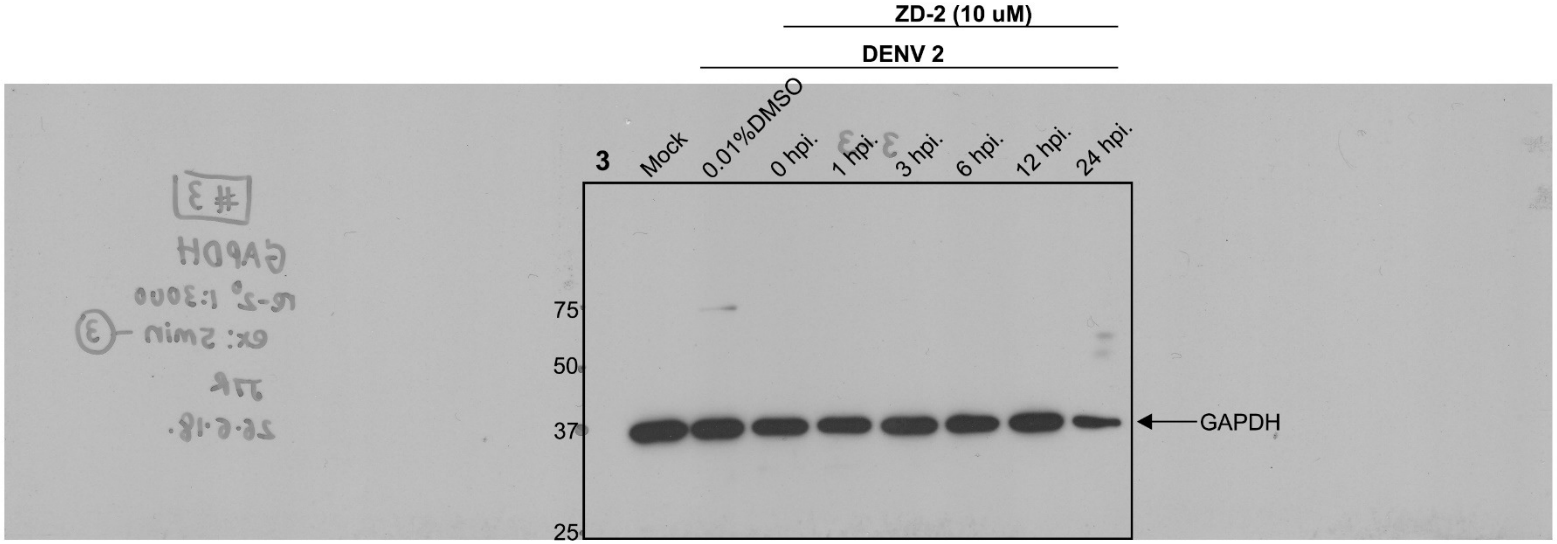
ZD-2 (10 uM)
DENV 2
3
Mock
0.01%DMSO
0 hpi.
1 hpi.
3 hpi.
6 hpi.
12 hpi.
24 hpi.
75
50
37
25
GAPDH

ZD-3

ZD-3: NS5

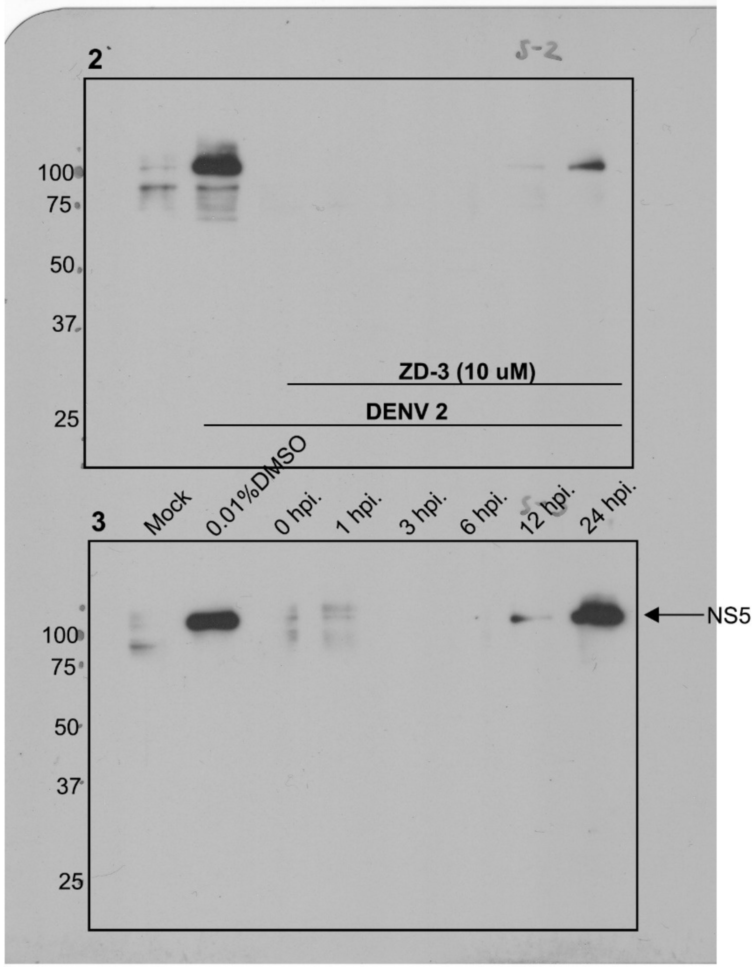
2
5-2
100
75
50
37
25
ZD-3 (10 uM)
DENV 2
3
Mock
0.01%DMSO
0 hpi.
1 hpi.
3 hpi.
6 hpi.
12 hpi.
24 hpi.
100
75
50
37
25
NS5

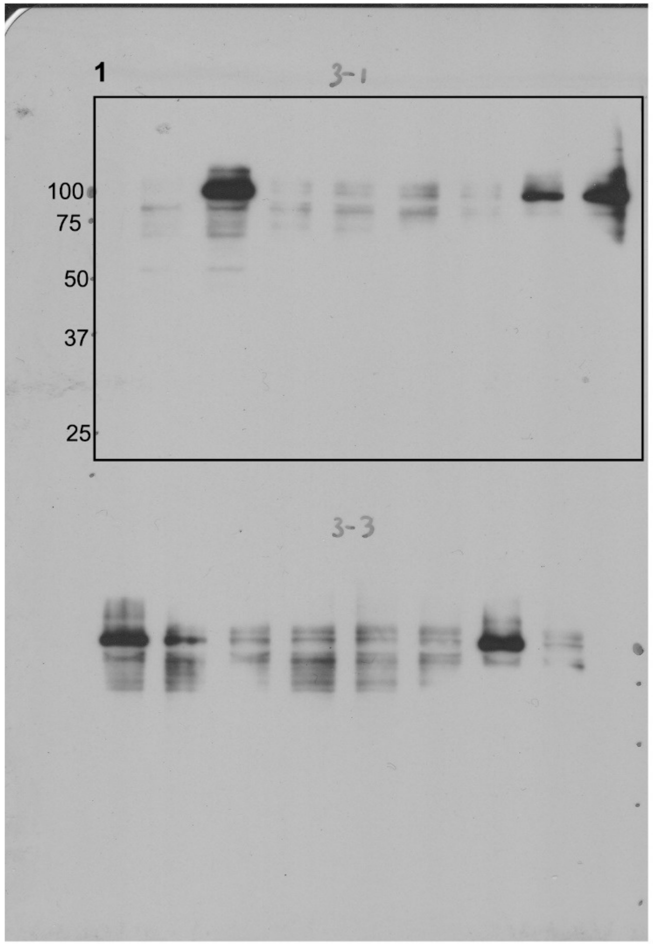
1
3-1
100
75
50
37
25
3-3

ZD-3: NS3

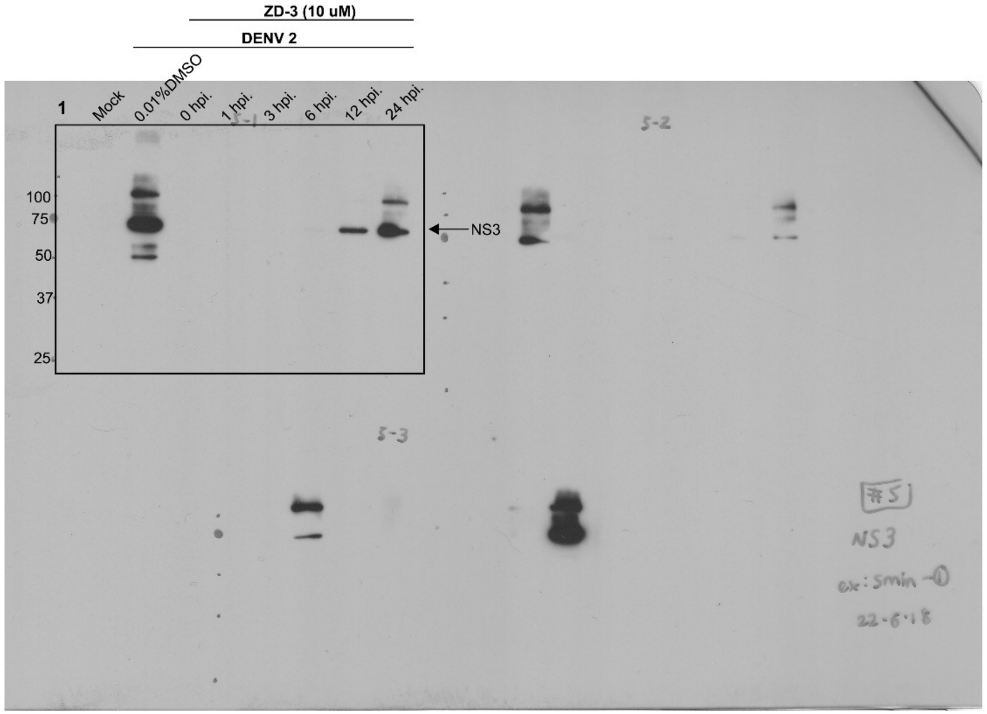
ZD-3 (10 uM)
DENV 2
1
Mock
0.01%DMSO
0 hpi.
1 hpi.
3 hpi.
6 hpi.
12 hpi.
24 hpi.
100
75
50
37
25
NS3

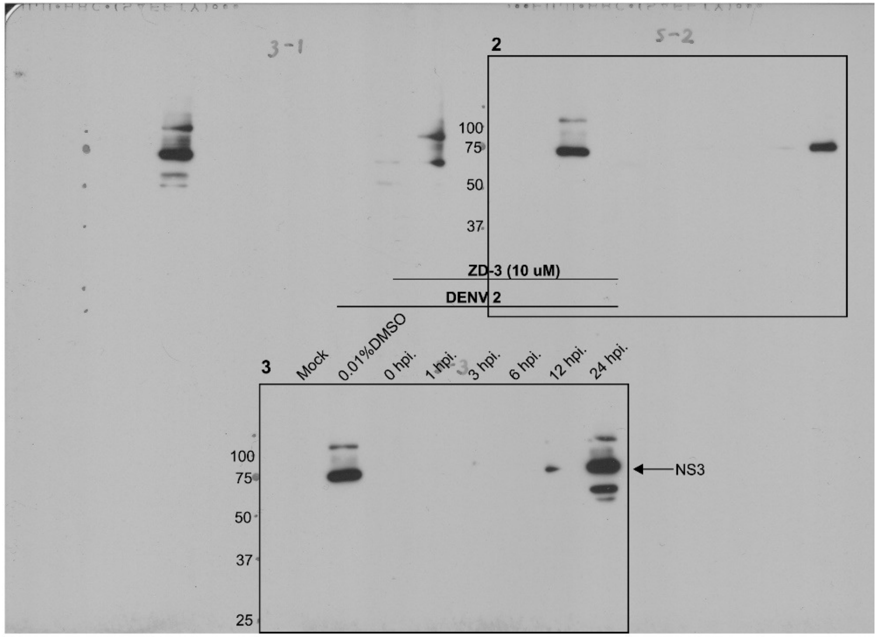
2
100
75
50
37
ZD-3 (10 uM)
DENV 2
3
Mock
0.01%DMSO
0 hpi.
1 hpi.
3 hpi.
6 hpi.
12 hpi.
24 hpi.
100
75
50
37
25
NS3

ZD-3: E

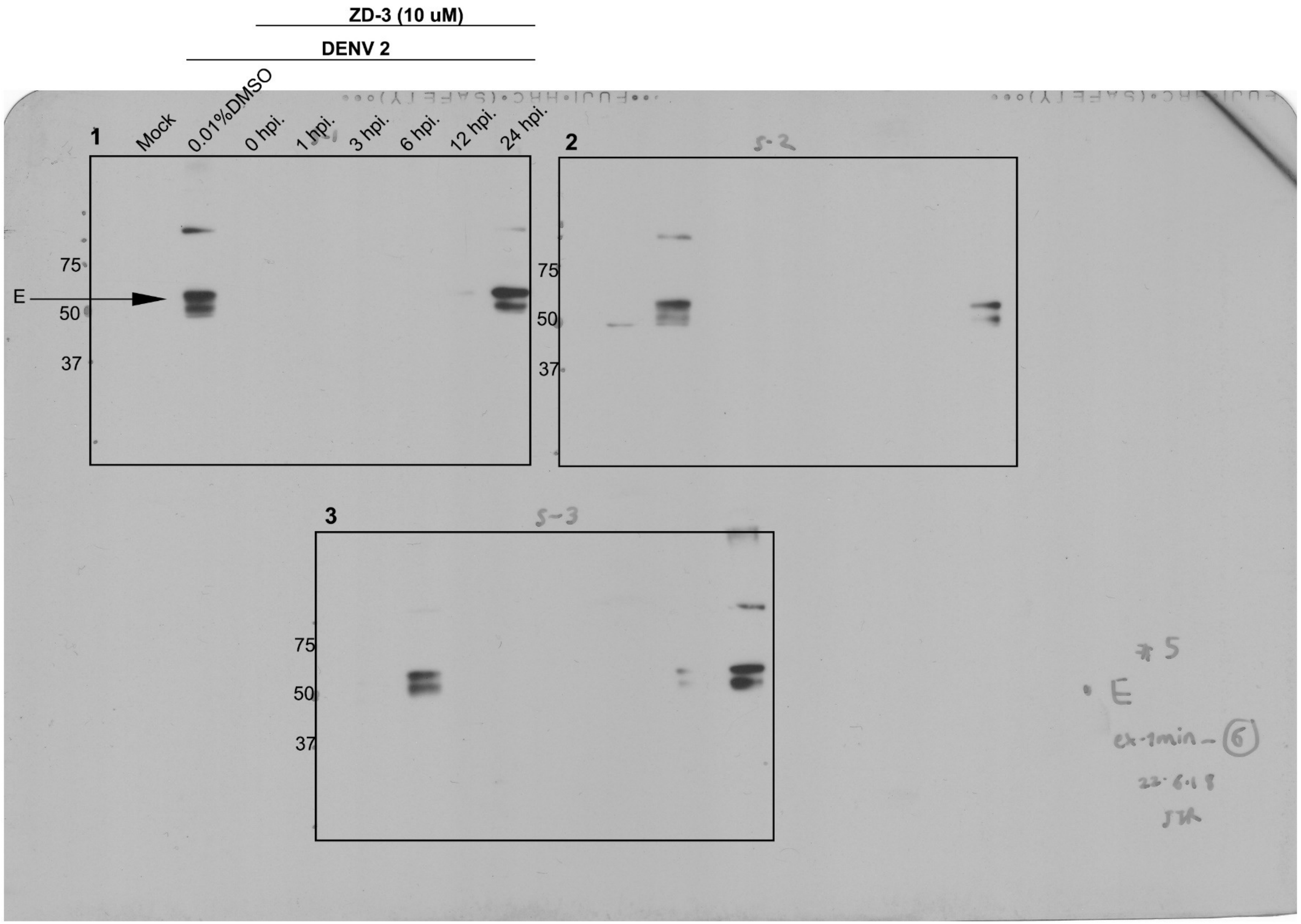

ZD-3 (10 uM)
DENV 2
Mock
0.01%DMSO
0 hpi.
1 hpi.
3 hpi.
6 hpi.
12 hpi.
24 hpi.
1
2
3
75
50
37
E
s-2
s-3
#5
E
ex-1min-6
22.6.18

ZD-3: NS1

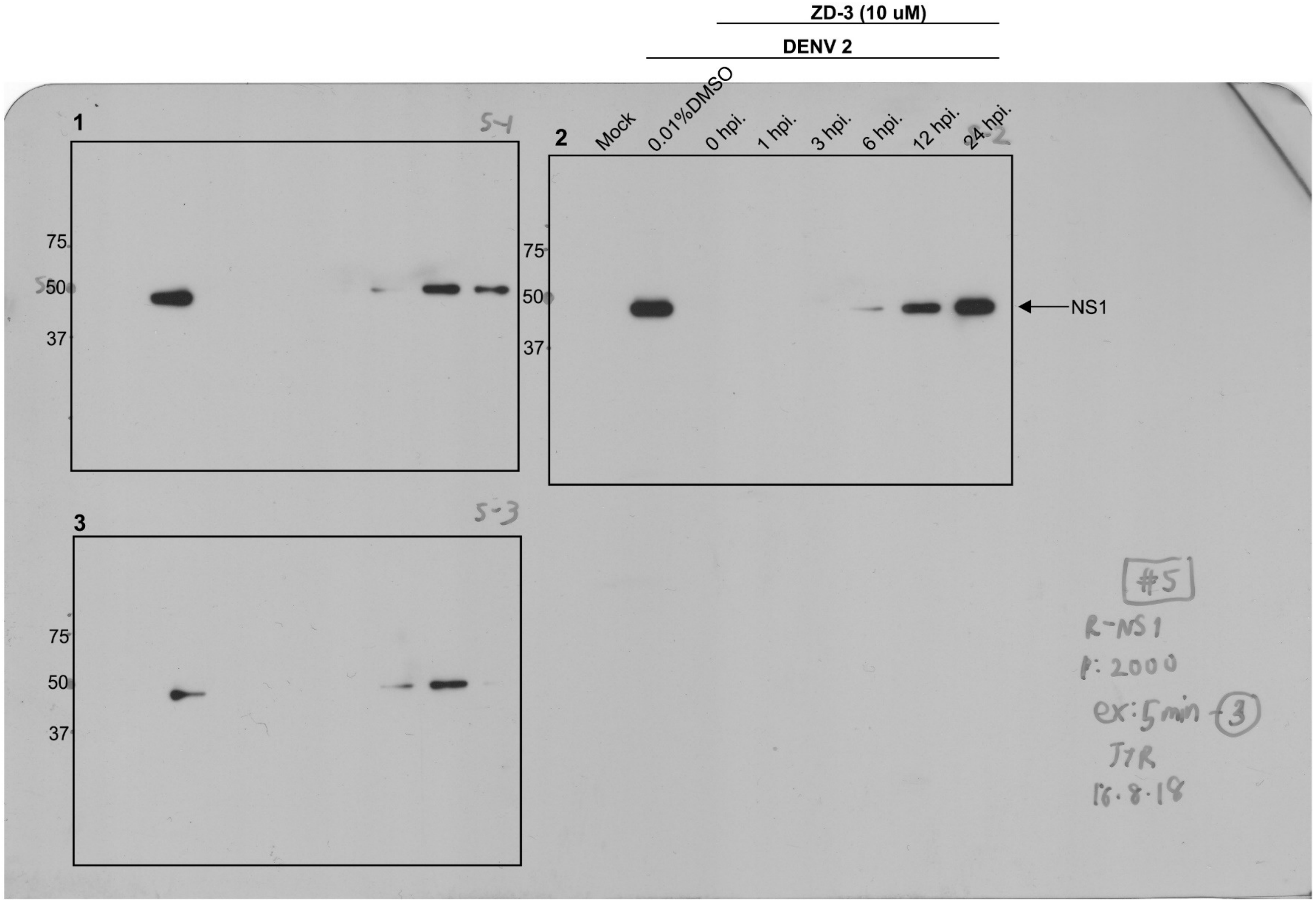

ZD-3 (10 uM)
DENV 2
1
2
3
Mock
0.01%DMSO
0 hpi.
1 hpi.
3 hpi.
6 hpi.
12 hpi.
24 hpi.
75
50
37
NS1
5-4
5-3
#5
R-NS1
1:2000
ex:5min-3
JTR
16.8.18

ZD-3: GAPDH

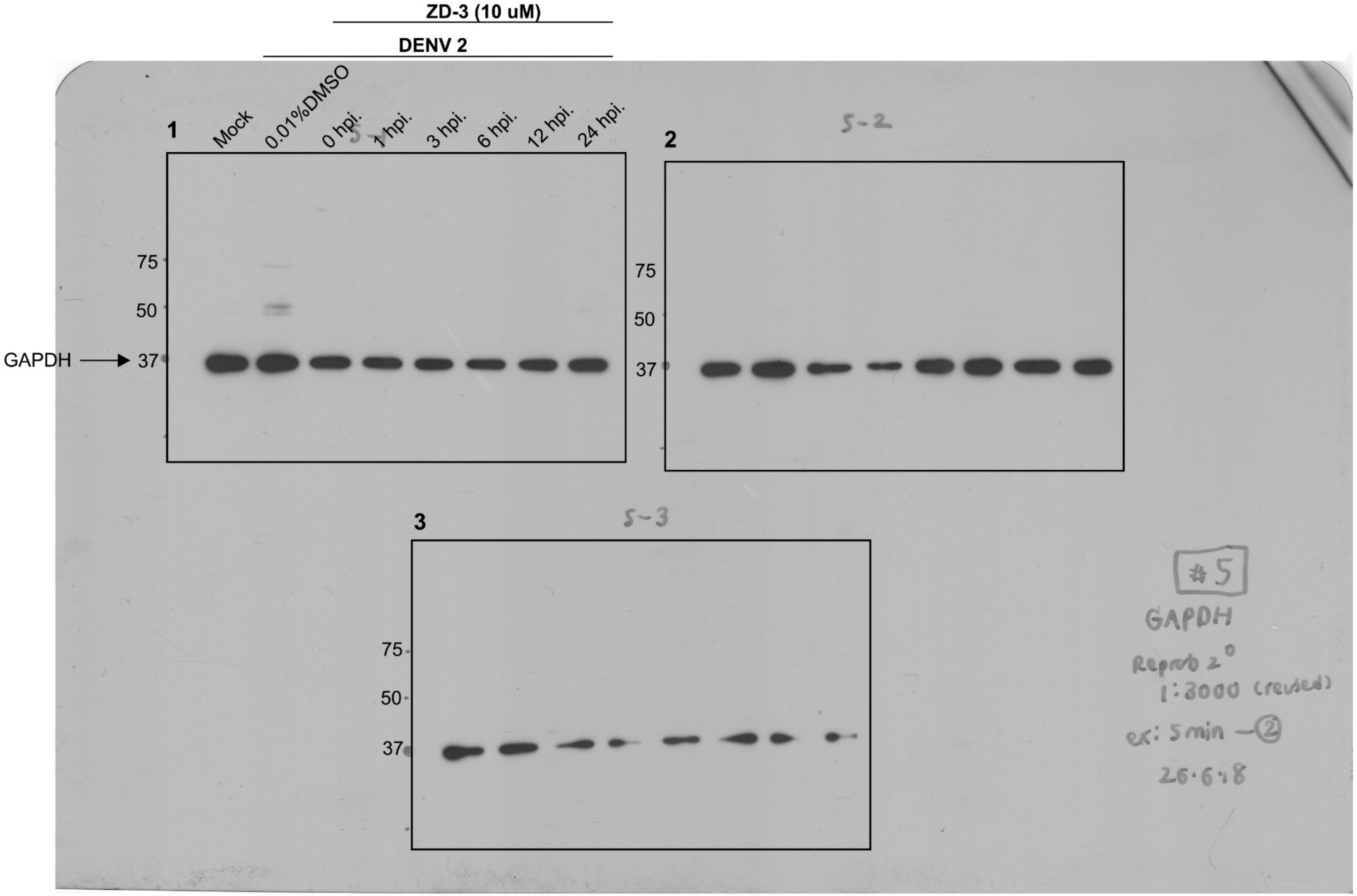

ZD-3 (10 uM)
DENV 2
1
Mock
0.01%DMSO
0 hpi.
1 hpi.
3 hpi.
6 hpi.
12 hpi.
24 hpi.
75
50
GAPDH
37
2
S-2
75
50
37
3
S-3
75
50
37
#5
GAPDH
Reprob 2°
1:3000 (revised)
ex: 5min —②
26.6.18

ZD-5

ZD-5: NS5

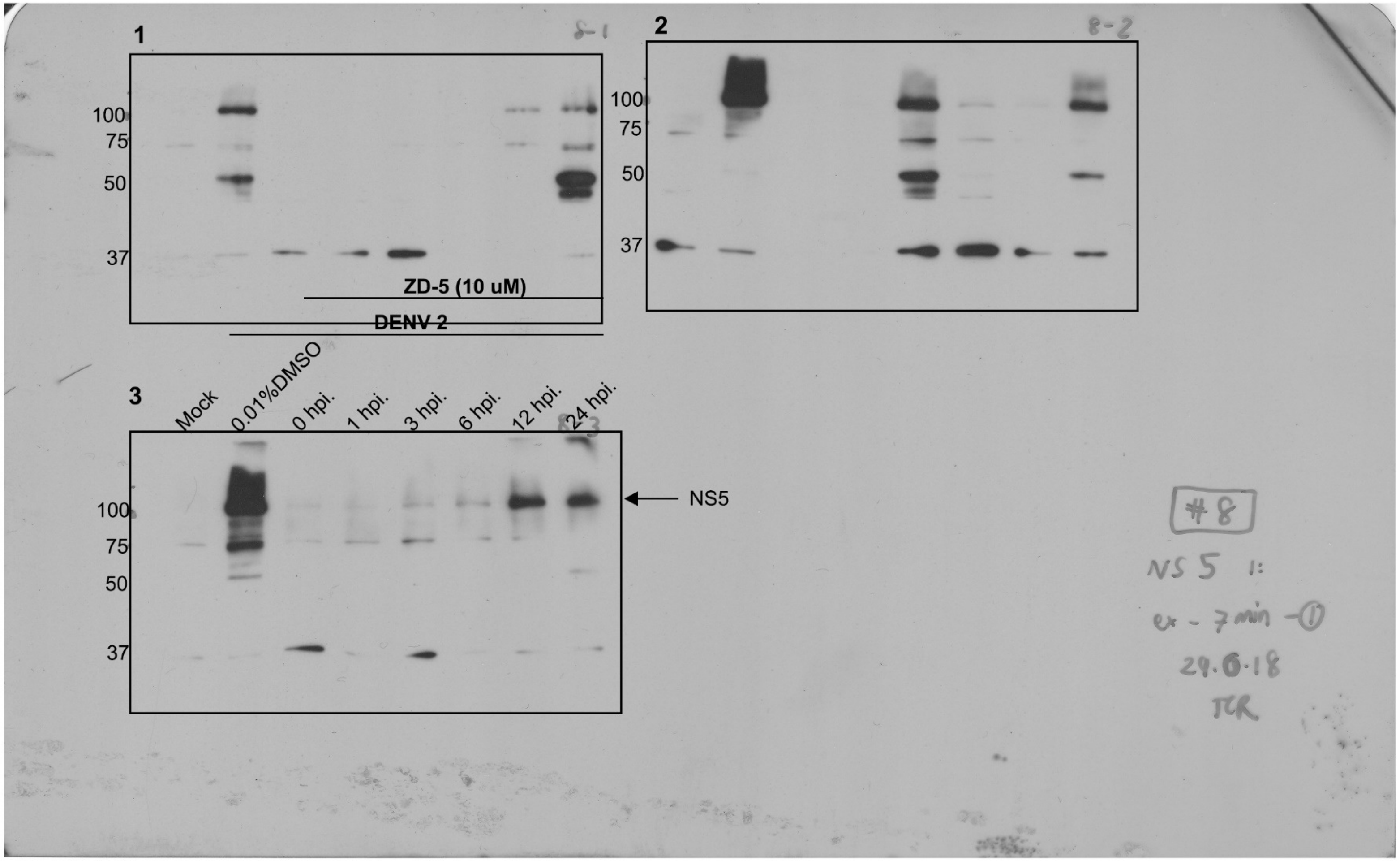
1
8-1
100
75
50
37
ZD-5 (10 uM)
DENV 2
2
8-2
100
75
50
37
3
Mock
0.01%DMSO
0 hpi.
1 hpi.
3 hpi.
6 hpi.
12 hpi.
24 hpi.
100
75
50
37
NS5
#8
NS 5 1:
ex - 7 min - ①
29.6.18

ZD-5: NS3

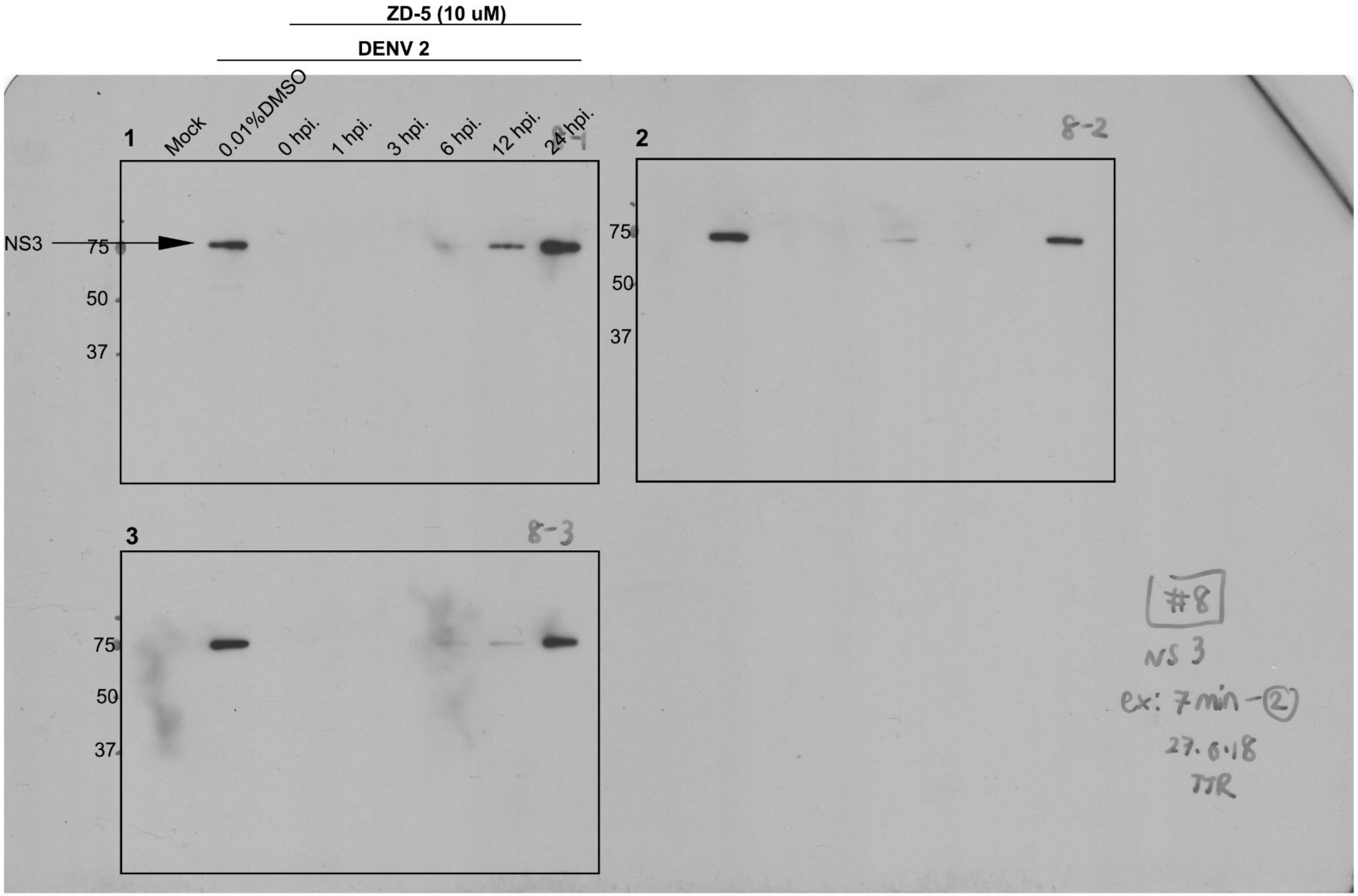

ZD-5 (10 uM)
DENV 2
1
Mock
0.01%DMSO
0 hpi.
1 hpi.
3 hpi.
6 hpi.
12 hpi.
24 hpi.
NS3
75
50
37
2
8-2
75
50
37
3
8-3
75
50
37
#8
NS 3
ex: 7min - ②
27.6.18
TJR

ZD-5: E

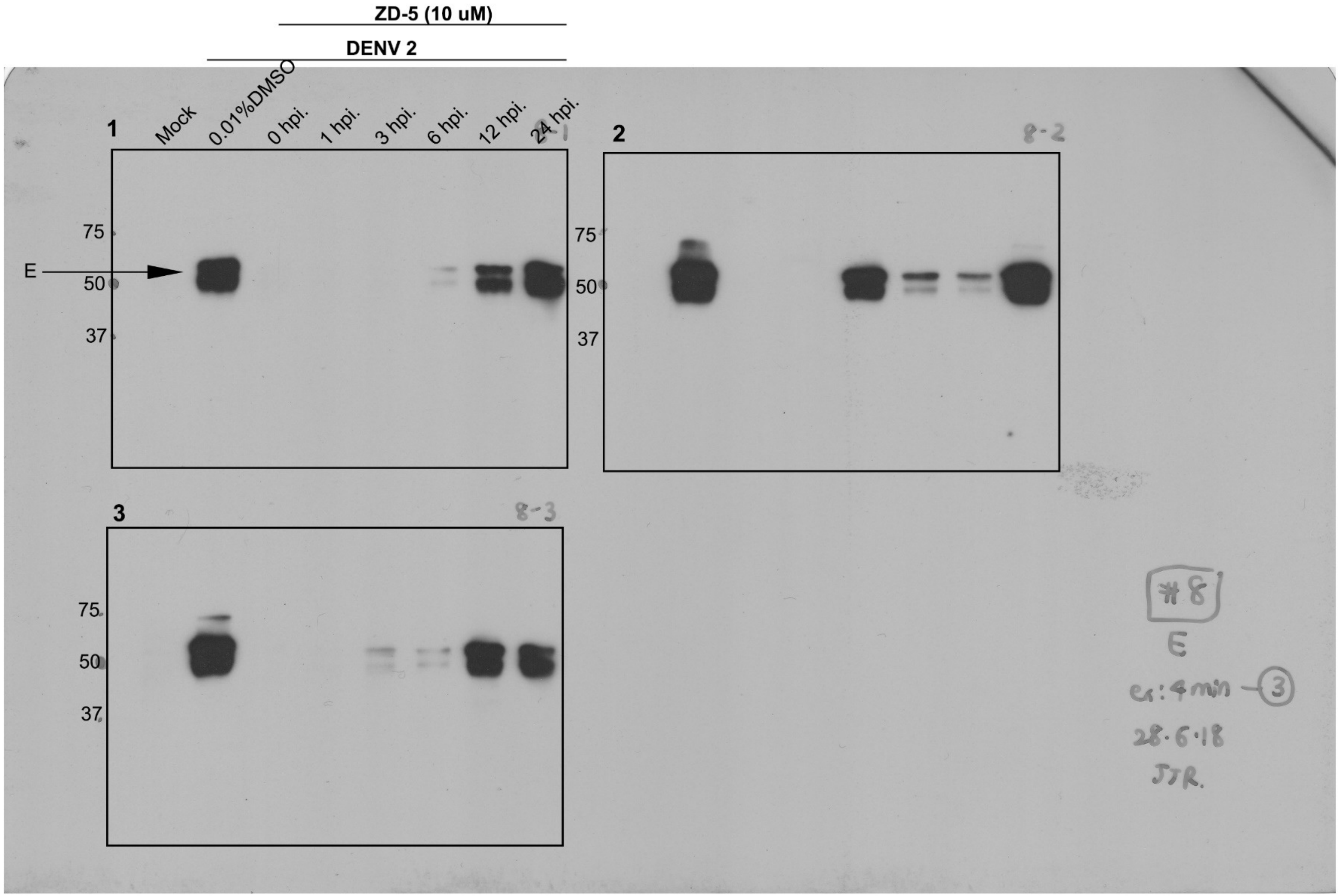
ZD-5 (10 uM)
DENV 2
1
Mock
0.01%DMSO
0 hpi.
1 hpi.
3 hpi.
6 hpi.
12 hpi.
24 hpi.
2
3
75
50
37
E
8-2
8-3
#8
E
28·6·18

ZD-5: NS1

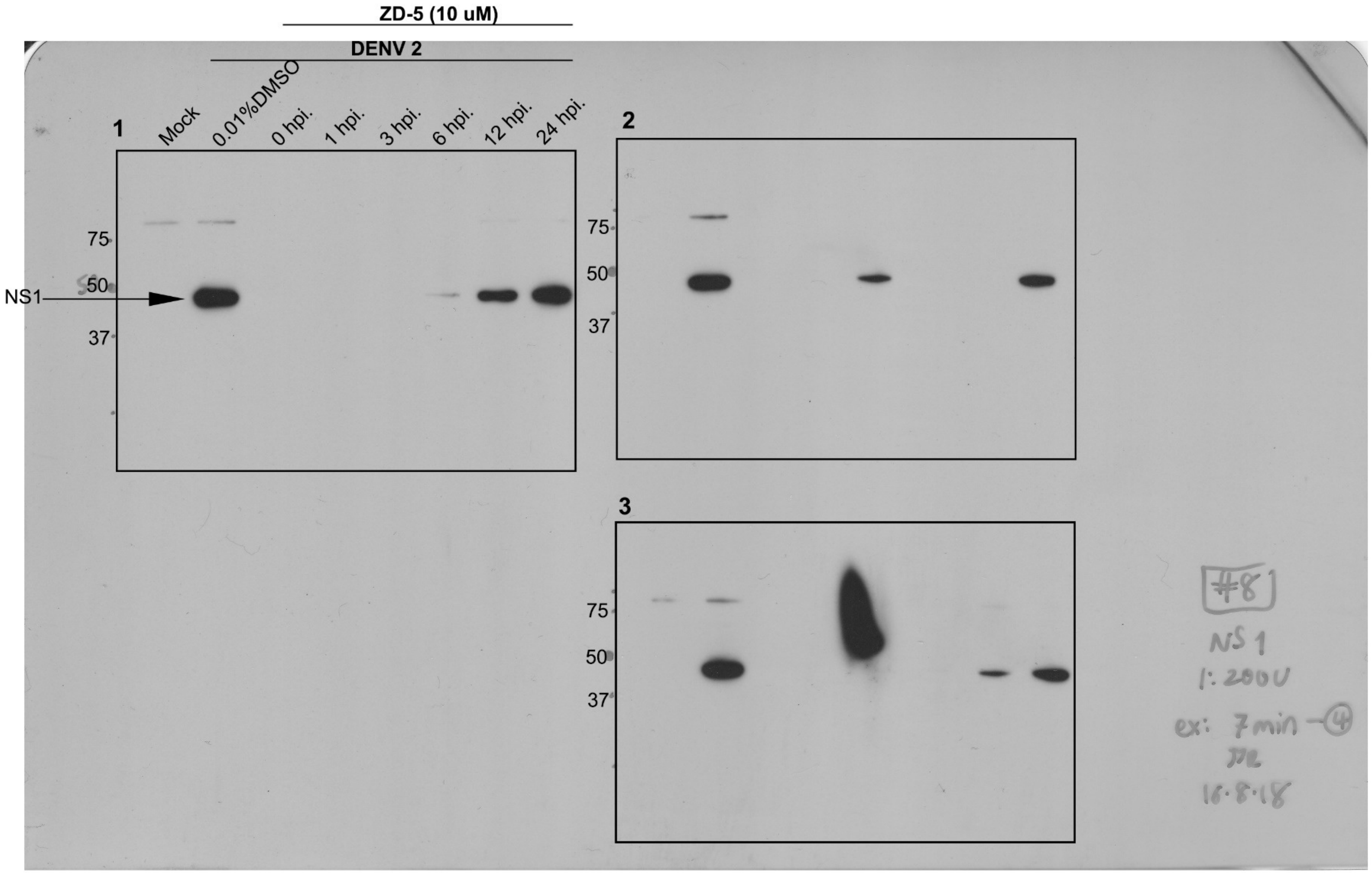

ZD-5 (10 uM)
DENV 2
1
Mock
0.01%DMSO
0 hpi.
1 hpi.
3 hpi.
6 hpi.
12 hpi.
24 hpi.
75
50
37
NS1
2
75
50
37
3
75
50
37
#8
NS 1
1: 2000
ex: 7min
16.8.18

ZD-5: GAPDH

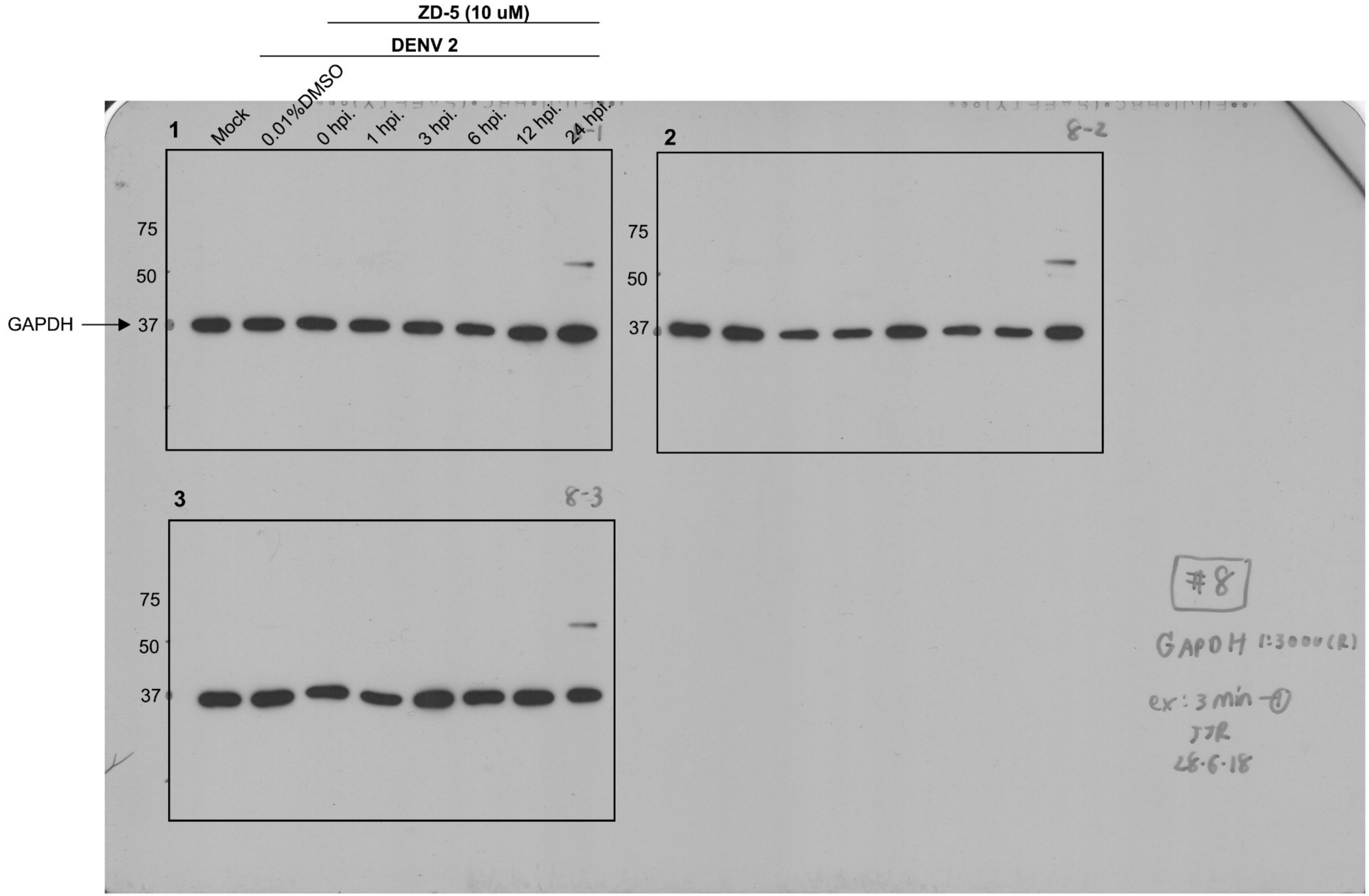
ZD-5 (10 uM)
DENV 2
1
Mock
0.01%DMSO
0 hpi.
1 hpi.
3 hpi.
6 hpi.
12 hpi.
24 hpi.
75
50
37
GAPDH
2
8-2
3
8-3
#8
GAPDH 1:3000 (R)
ex: 3 min -①
JJR
28-6-18

ZD-6

ZD-6: NS5

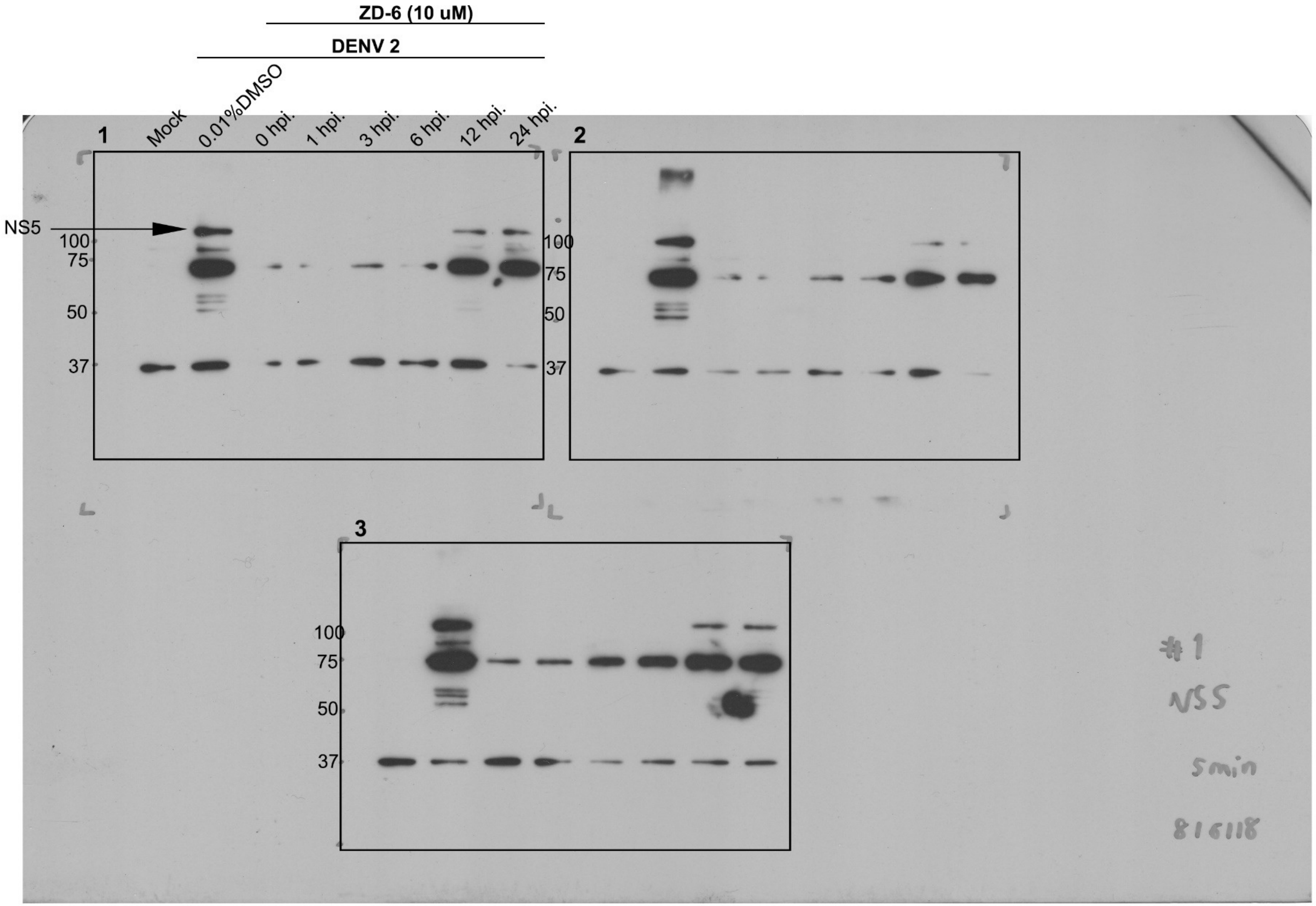
ZD-6 (10 uM)
DENV 2
Mock
0.01%DMSO
0 hpi.
1 hpi.
3 hpi.
6 hpi.
12 hpi.
24 hpi.
1
2
3
NS5
100
75
50
37
#1
NS5
5min
8/6/18

ZD-6: NS3

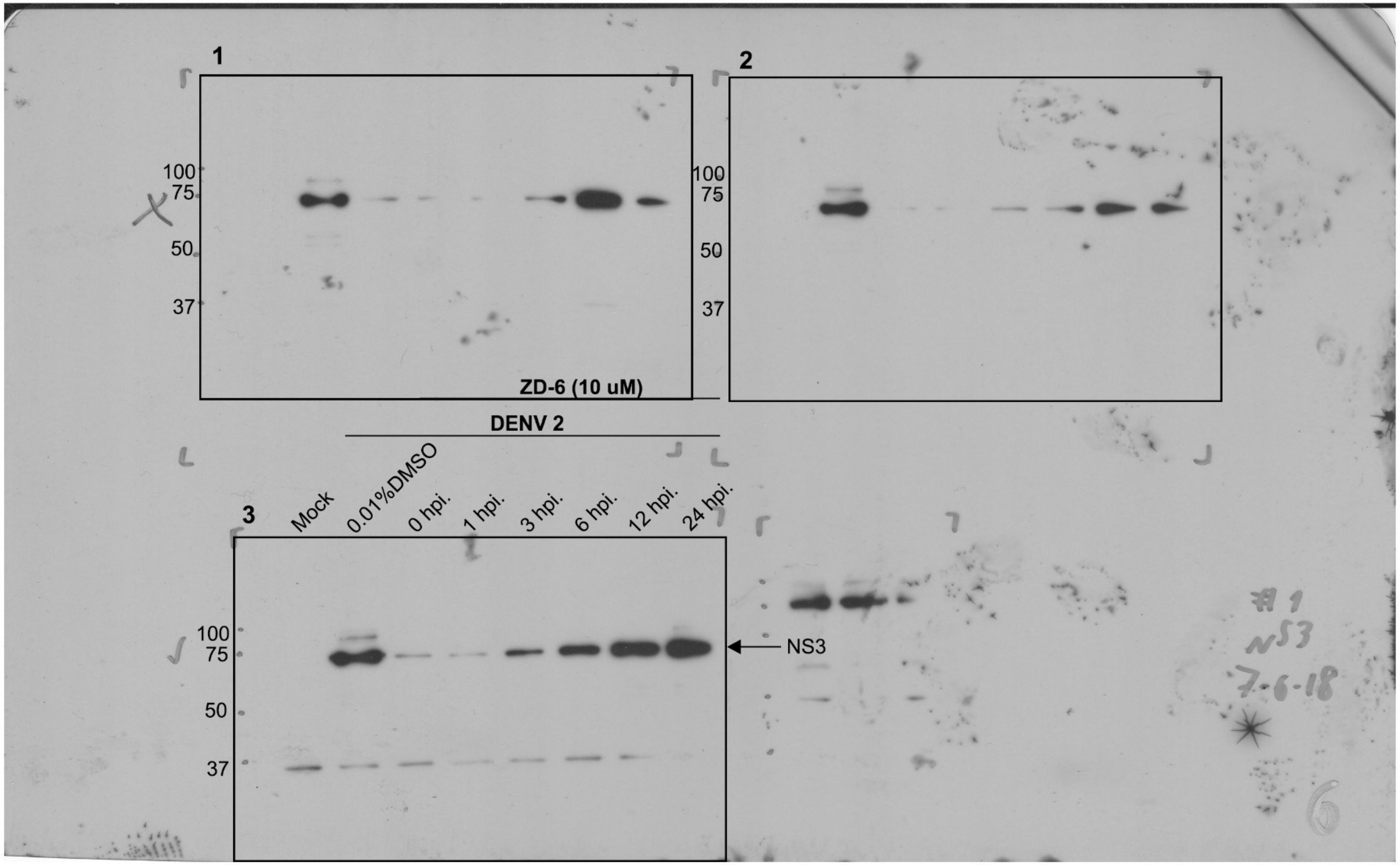
1
2
3
100
75
50
37
ZD-6 (10 uM)
DENV 2
Mock
0.01%DMSO
0 hpi.
1 hpi.
3 hpi.
6 hpi.
12 hpi.
24 hpi.
NS3
7-6-18

ZD-6: E

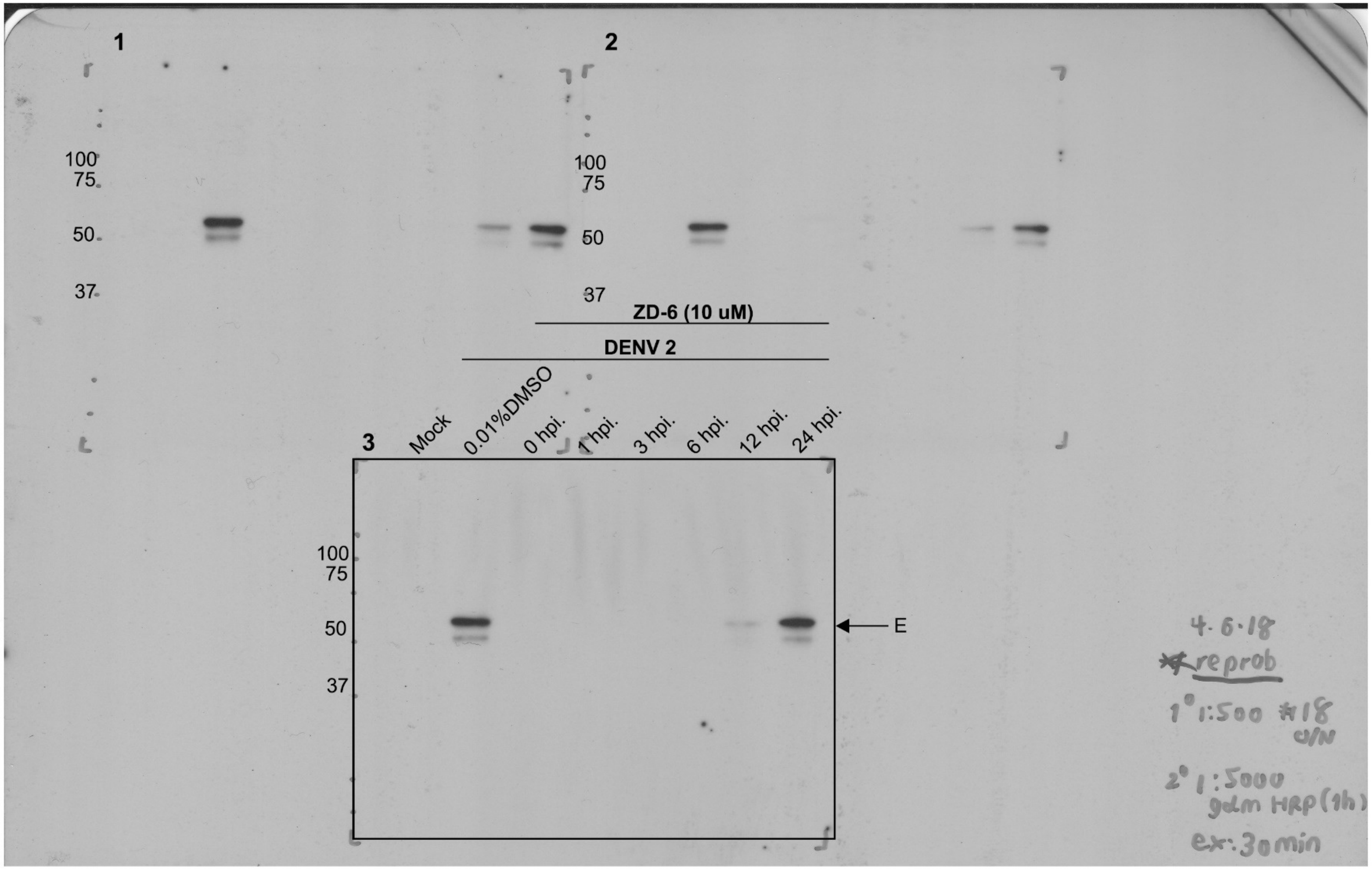
1
2
100
75
50
37
ZD-6 (10 uM)
DENV 2
3
Mock
0.01%DMSO
0 hpi.
1 hpi.
3 hpi.
6 hpi.
12 hpi.
24 hpi.
E
4.6.18
reprob
1° 1:500 #18 O/N
2° 1:5000 gdm HRP (1h)
ex: 30min

ZD-6: NS1

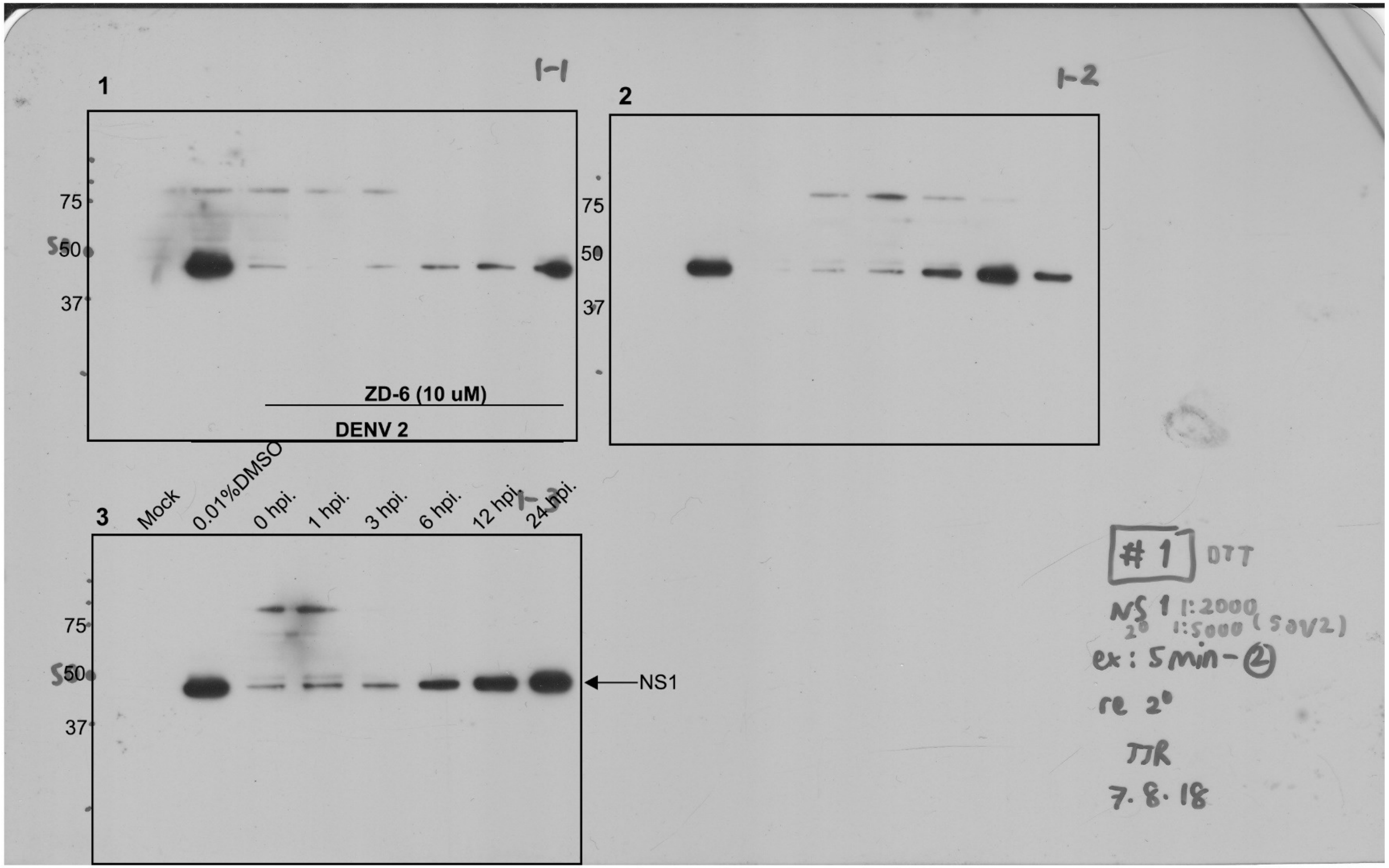
1
1-1
75
50
37
ZD-6 (10 uM)
DENV 2
2
1-2
75
50
37
3
Mock
0.01%DMSO
0 hpi.
1 hpi.
3 hpi.
6 hpi.
12 hpi.
24 hpi.
1-3
75
50
37
NS1
#1 DTT
NS 1 1:2000
2° 1:5000 (SOV2)
ex: 5min-②
re 2°
TJR
7.8.18

ZD-6: GAPDH

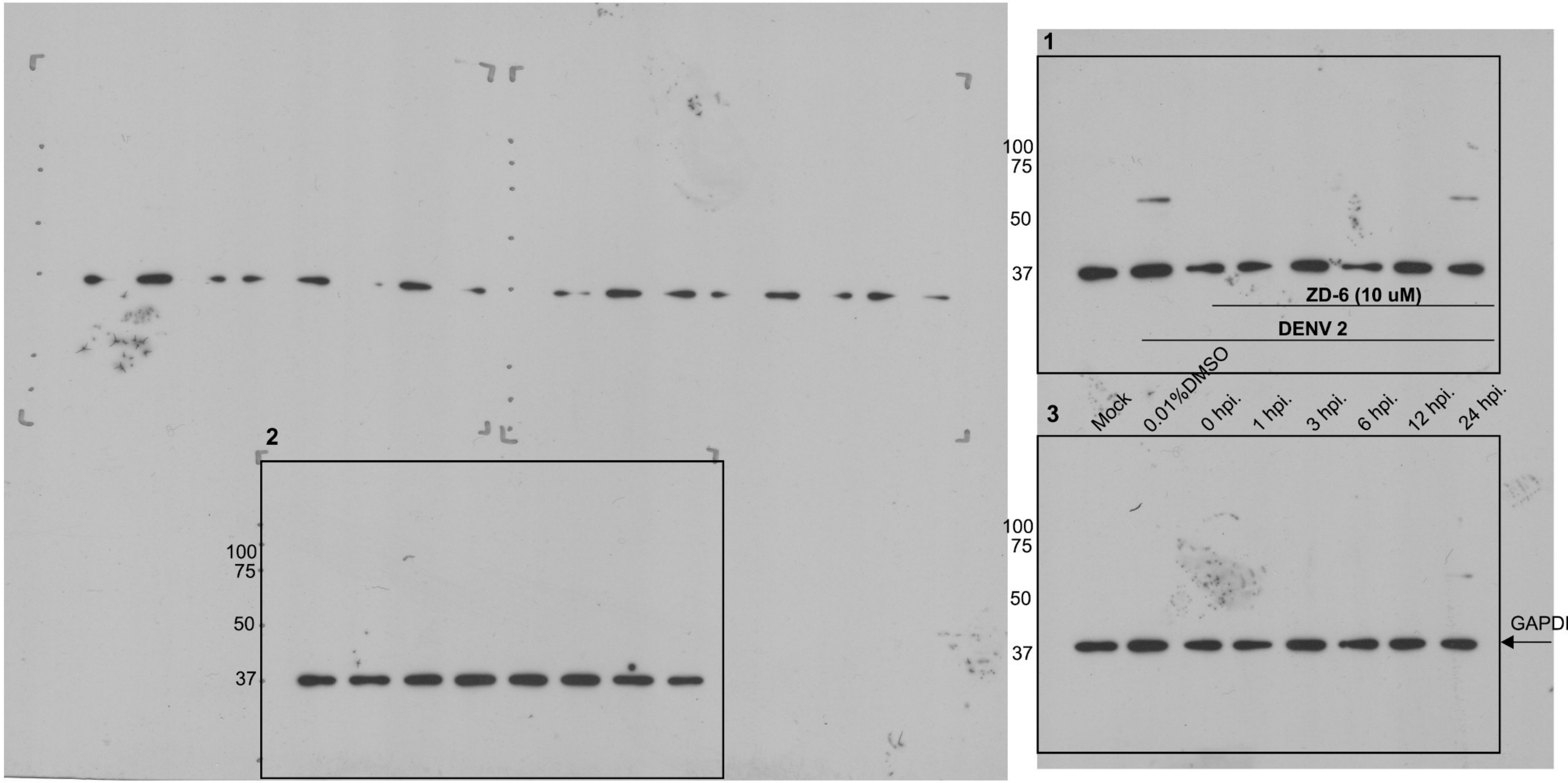
1
2
3
100
75
50
37
ZD-6 (10 uM)
DENV 2
Mock
0.01%DMSO
0 hpi.
1 hpi.
3 hpi.
6 hpi.
12 hpi.
24 hpi.
GAPDH
